# B cell subsets contribute to myocardial protection by inducing neutrophil apoptosis after ischemia and reperfusion

**DOI:** 10.1172/jci.insight.167201

**Published:** 2024-02-22

**Authors:** Fangyang Huang, Jialiang Zhang, Hao Zhou, Tianyi Qu, Yan Wang, Kexin Jiang, Yutong Liu, Yuanning Xu, Mao Chen, Li Chen

**Affiliations:** 1 Department of Cardiology,; 2 State Key Laboratory of Biotherapy and Cancer Center,; 3 Laboratory of Heart Valve Disease,; 4 West China School of Medicine, and; 5 Laboratory of Cardiovascular Diseases, Regenerative Medicine Research Center, West China Hospital, Sichuan University, Chengdu, China.

**Keywords:** Cardiology, Immunology, Apoptosis, Cardiovascular disease, Innate immunity

## Abstract

A robust, sterile inflammation underlies myocardial ischemia and reperfusion injury (MIRI). Several subsets of B cells possess the immunoregulatory capacity that limits tissue damage, yet the role of B cells in MIRI remains elusive. Here, we sought to elucidate the contribution of B cells to MIRI by transient ligation of the left anterior descending coronary artery in B cell–depleted or –deficient mice. Following ischemia and reperfusion (I/R), regulatory B cells are rapidly recruited to the heart. B cell–depleted or –deficient mice exhibited exacerbated tissue damage, adverse cardiac remodeling, and an augmented inflammatory response after I/R. Rescue and chimeric experiments indicated that the cardioprotective effect of B cells was not solely dependent on IL-10. Coculture experiments demonstrated that B cells induced neutrophil apoptosis through contact-dependent interactions, subsequently promoting reparative macrophage polarization by facilitating the phagocytosis of neutrophils by macrophages. The in vivo cardioprotective effect of B cells was undetectable in the absence of neutrophils after I/R. Mechanistically, ligand-receptor imputation identified FCER2A as a potential mediator of interactions between B cells and neutrophils. Blocking FCER2A on B cells resulted in a reduction in the percentage of apoptotic neutrophils, contributing to the deterioration of cardiac remodeling. Our findings unveil a potential cardioprotective role of B cells in MIRI through mechanisms involving FCER2A, neutrophils, and macrophages.

## Abstract 2

<p>FCER2A^+ ^B cells can mitigate the cardiac inflammatory response and attenuate myocardial injury after myocardial ischemia and reperfusion injury by inducing neutrophil&nbsp;apoptosis in a contact-dependent manner.</p>

## Introduction

Despite advancements in myocardial reperfusion strategies, the risk of mortality associated with acute myocardial infarction (MI) persists at a stubbornly high level. Myocardial ischemia and reperfusion injury (MIRI) significantly contributes to cardiomyocyte death and can account for nearly half of the final infarct size (1). Several aspects of pathological processes are involved in MIRI, with robust sterile inflammation being a common feature. An early proinflammatory response following myocardial ischemia is imperative for the clearance of damaged cells, facilitating the transition to subsequent reparative programs (2). However, if it is intense, prolonged, or insufficiently suppressed, early inflammation activation will amplify tissue damage and give rise to subsequent adverse cardiac remodeling. Therefore, maintaining a dynamic equilibrium between proinflammation and antiinflammation has emerged as a pivotal strategy to mitigate MIRI.

B cells are traditionally regarded as proinflammatory antibody-producing cells, but recent studies revealed considerable phenotypic and functional diversity within the peripheral B cell compartment. Several subsets of B cells exhibit immunoregulatory capacities, playing a role in restraining autoreactive diseases and limiting tissue damage. Among these, regulatory B cells (Bregs) stand out as a subset that contributes to immune homeostasis following tissue injury. This contribution often involves the production of cytokines such as IL-10 and TGF-β, or adenosine, as well as cell-cell contact interaction through molecules like PD-L1 or FASL (3). In contrast to regulatory T cells (Tregs), Bregs lack a cell-specific marker or identified transcription factor. Bregs exhibit considerable phenotypic variability, with a murine study highlighting their distinctive feature of an increased propensity to produce IL-10 (4). Meanwhile, in comparison with T cells, B cells are equipped with numerous Toll-like receptors (TLRs) that enable them to promptly sense damage-associated molecular patterns (DAMPs) like mitochondrial DNA, S100A8/9, and HMGB1 in microenvironments. This capability allows B cells to swiftly respond to tissue injury. One study has reported that B cells, particularly those producing IL-10, predominantly regulate disease initiation, while Tregs reciprocally inhibit the late phase of disease (5).

Myocardial ischemic injury encompasses permanent ligation injury and ischemia-reperfusion (I/R) injury. In the context of the mouse permanent myocardial infarction (MI) model, previous studies have employed diverse strategies to interfere with B cells and targeted various subsets. Despite the distinct approaches utilized, these studies collectively provide valuable insights into the complex involvement of B cells in the context of permanent MI (6–8). In the revascularization era, the MIRI model offers a more realistic simulation of the clinical pathophysiological state of myocardial ischemia. As highlighted earlier, the early burst of proinflammatory response following myocardial I/R significantly contributes to infarction size. Consequently, early control of inflammation is crucial for attenuating MIRI. Clinical evidence has demonstrated impaired regulatory function and a decreased number of Bregs in the peripheral blood of patients with coronary artery disease (9–11). The adoptive transfer of Bregs has been shown to significantly reduce circulating leukocyte numbers and inflammatory monocytes in LDLR^–/–^ mice, which are susceptible to atherosclerosis (11). However, the data regarding the effect of Bregs on MIRI are scarce. Accordingly, the present study aims to elucidate the contribution of B cells to MIRI.

## Results

### The infiltration of B cells and Bregs after myocardial I/R.

**Figure 1:**
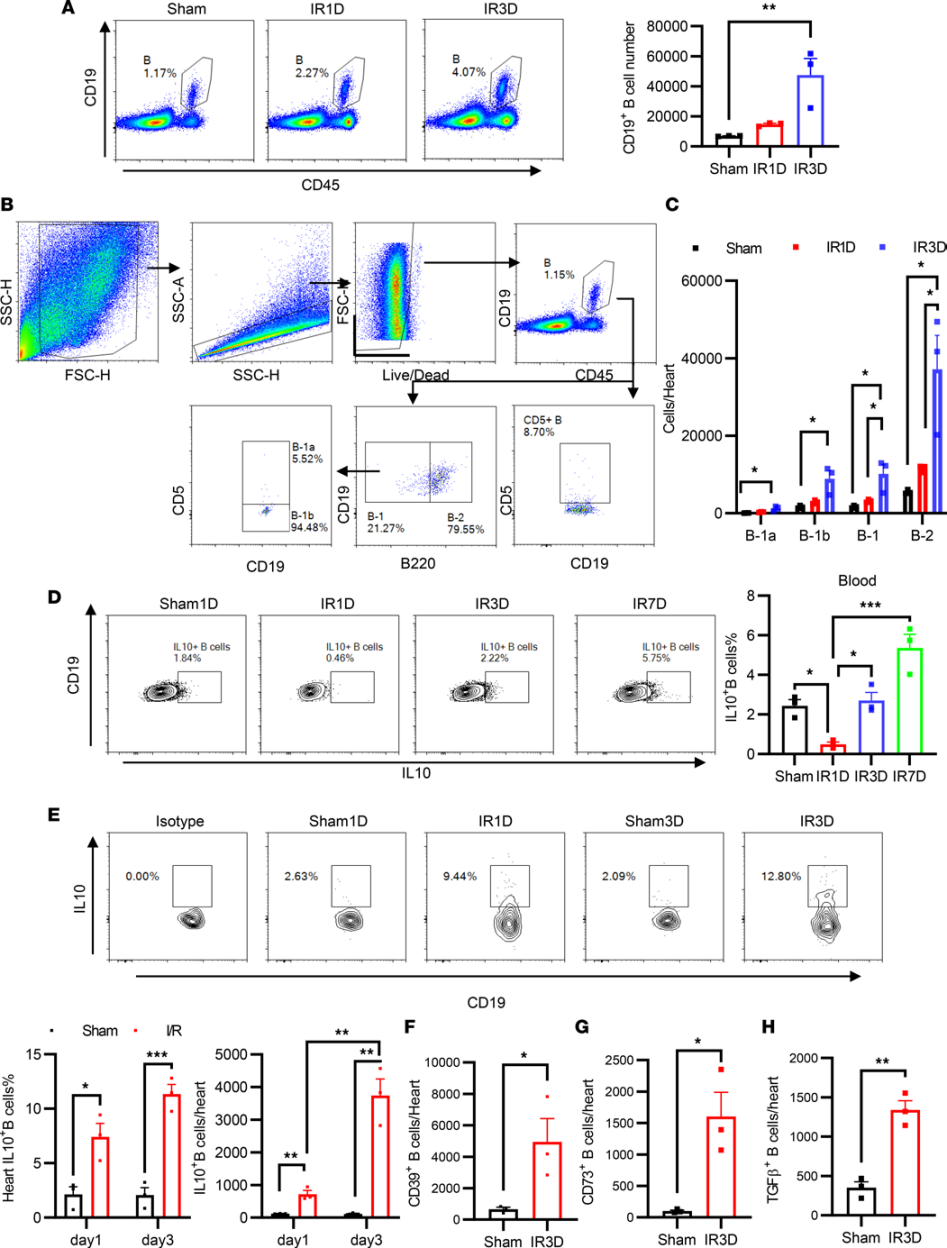
The infiltration of B cells and Bregs into the heart after myocardial I/R. (**A**) Representative images for flow cytometric analysis and flow cytometry–based quantification of the number of CD19^+^ B cells in digested heart tissues of mice before and 1 and 3 days after myocardial I/R. (**B**) Gating strategies and (**C**) quantification of the number of B cell subsets in the heart. (**D**) Quantification of the percentage of circulating IL-10^+^ B cells before and 1, 3, and 7 days after myocardial I/R. (**E**) Representative images for flow cytometric analysis and flow cytometry–based quantification of percentage and the number of IL-10^+^ B cells in digested heart tissue. (**F**–**H**) Quantification of the number of CD39^+^ B cells, CD73^+^ B cells, and TGF-β^+^ B cells of digested heart tissue before and 3 days after myocardial I/R. These experiments were independently replicated at least twice. I/R, ischemia and reperfusion. Data are presented as mean ± SEM. *P < 0.05; **P < 0.01; ***P < 0.001 by 1-way ANOVA test followed by Tukey’s post hoc test (**A** and **C**–**E**); differences between 2 groups were compared by unpaired, 2-tailed *t* test (**F**–**H**).

To examine whether B cells are present in the heart after myocardial I/R, mice were subjected to transient coronary artery ligation, simulating the situation of patients with acute MI undergoing reperfusion therapy. On 1 and 3 days after myocardial I/R, the myocardial CD19^+^ cells were quantified by flow cytometry (Figure 1A). CD19^+^ B cells began to accumulate in the heart as early as 24 hours after I/R, and the number of myocardial B cells further increased 72 hours after myocardial I/R. Mature B cells are classified as B-1 and B-2 cells. B-1 B cells were further subdivided into B-1a and B-1b B cells based on the expression of CD5. We assessed myocardial B cell compartments following I/R using both a widely employed strategy, as reported by Lan et al. (Figure 1B) (7), and a modified gating approach (Supplemental Figure 1A; supplemental material available online with this article; https://doi.org/10.1172/jci.insight.167201DS1). Both strategies consistently demonstrated an increased absolute number of all B cell subsets 3 days after I/R, with a predominant presence of B-2 cells (Figure 1C and Supplemental Figure 1B). IL-10–producing B cells are recognized for their immunoregulatory capacity. To explore this, we investigated the levels of blood and myocardial IL-10–producing B cells following I/R. The frequency of IL-10–producing B cells in the blood exhibited a transient decrease on day 1 after I/R, followed by a subsequent expansion peaking on day 7 (Figure 1D). Both the percentage and total number of IL-10–producing B cells in the heart gradually increased after I/R (Figure 1E). Recognizing the challenges associated with the enumeration of IL-10–producing B cells due to the intrinsic instability of IL-10 mRNA and transient low level of IL-10 protein expression (12), we also examined the IL-10 expression of B cells using IL-10 reporter mice (Vert-X mice), and similar changes were observed (Supplemental Figure 2A). In addition, the immunoregulatory effect of B cells was also associated with the secretion of TGF-β1 and adenosine (13). We identified a significant increase in TGF-β1^+^, CD39^+^, and CD73^+^ B cells in the heart after I/R (Figure 1, F–H, and Supplemental Figure 2B). These observations collectively indicate an increase in B cells and Breg subsets in the heart following myocardial I/R.

### Selective depletion of B cells or B cell deficiency aggravates tissue damage and cardiac remodeling following myocardial I/R and B cells protect against MIRI through an IL-10–independent mechanism.

**Figure 2:**
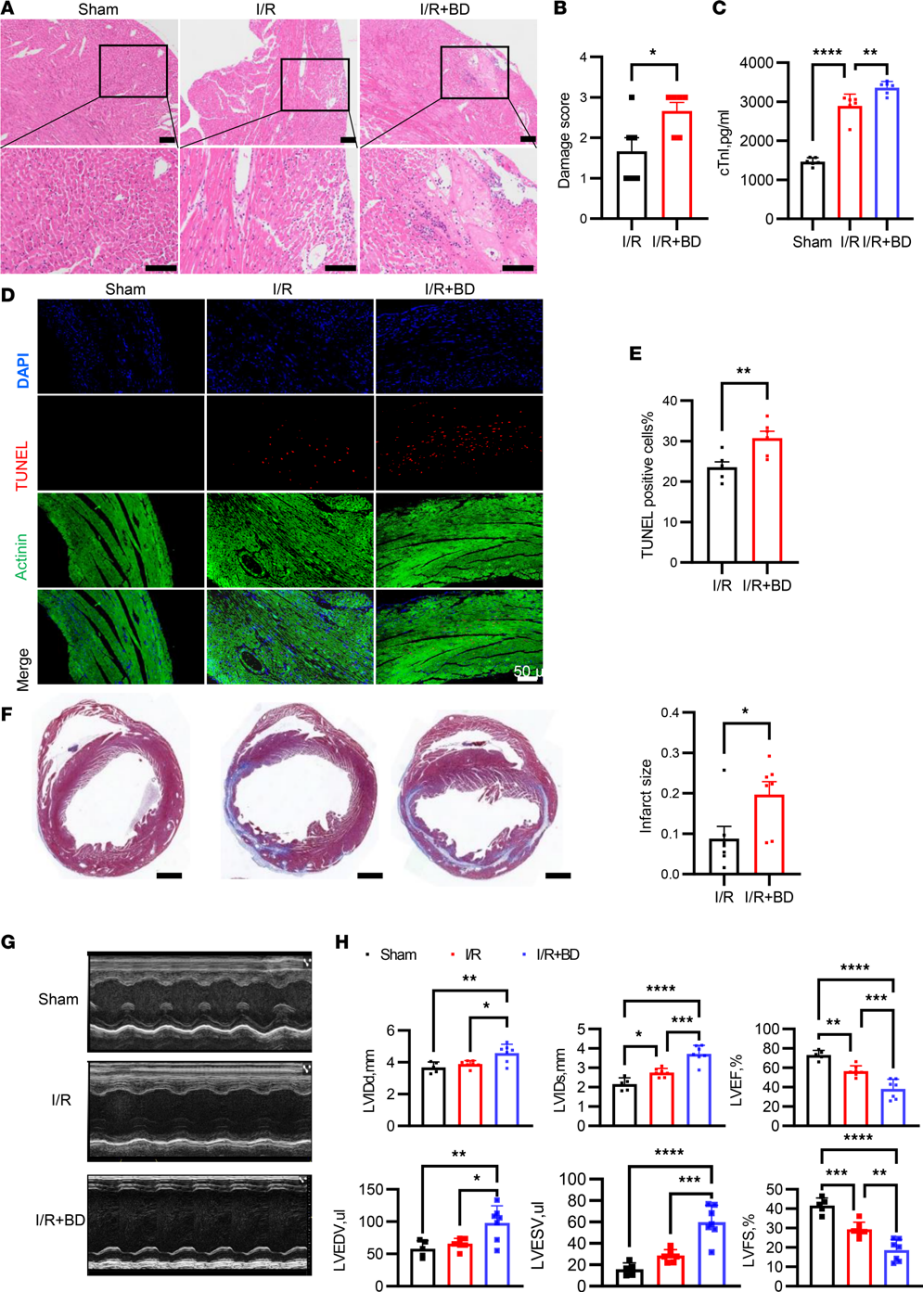
Selective B cell depletion accentuated tissue damage and deteriorated cardiac remodeling and cardiac function after myocardial I/R. (**A**) Representative images of H&E staining of heart slices in all groups. Scale bars: 50 μm. (**B**) Damage score evaluation of heart slice from mice with or without B cell depletion 1 day after myocardial I/R. (**C**) The measurement of serum troponin I in each group 1 day after MIRI. (**D** and **E**) Representative photos of TUNEL staining of heart slices and quantification in each group. Scale bar: 50 μm. (**F**) Scar size 14 days after myocardial I/R. Tissue slices were stained with Masson’s trichrome. Scale bars: 1000 μm. The infarct size was determined by calculating the ratio of the collagen area to the left ventricular area. (**G**) Representative M-mode echocardiography 14 days after myocardial I/R. (**H**) Measurement of LVIDd, LVIDs, LVEF, LVEDV, LVID, and LVFS in each group. Data are presented as mean ± SEM. *P < 0.05; **P < 0.01; ***P < 0.001; ****P < 0.0001 by unpaired, 2-tailed *t* test (**B**, **E**, and **F**) or 1-way ANOVA test followed by Tukey’s post hoc test (**C** and **H**). BD, B cell depletion; I/R, ischemia and reperfusion; LVIDd, left ventricular diastolic dimension; LVIDs, left ventricular systolic dimension; LVEF, left ventricular ejection fraction; LVFS, left ventricular fractional shortening; TUNEL, terminal deoxynucleotidyl transferase–mediated nick-end labeling.

The observed increase in B cells in the heart after I/R suggests their potential participation in the pathophysiological process of MIRI. To investigate the regulatory role of infiltrating B cells in MIRI, we employed 2 distinct approaches: anti-CD20–mediated depletion of B cells in mice and B cell–deficient (μMT) mice. The intravenous (i.v.) administration of mAb SA271G2 induced nearly complete depletion of B cells in both the heart and blood within 24 hours after injection (Supplemental Figure 3). Initially, we evaluated cardiac histopathological changes following I/R using a scoring system. Our observations revealed more pronounced myocardial damage and increased inflammatory cell infiltration in mice subjected to B cell depletion (Figure 2, A and B). Since the release of cardiac biomarkers reflects the extent of MIRI, we investigated whether the depletion of B cells influences the levels of cardiac troponin I (cTnI) following myocardial I/R. Selective depletion of B cells resulted in significantly increased cTnI (Figure 2C). Cardiac apoptotic cells, identified through the terminal deoxynucleotidyl transferase–mediated nick-end labeling (TUNEL) assay, were quantified in mice subjected to B cell depletion and those without. The number of TUNEL-positive cells significantly increased with B cell depletion treatment (Figure 2, D and E). Assessments of long-term cardiac function and adverse cardiac remodeling were conducted on day 14 after myocardial I/R. Cardiac contractile function worsened, and infarct size increased in the mice with B cell depletion (Figure 2, F–H).

**Figure 3:**
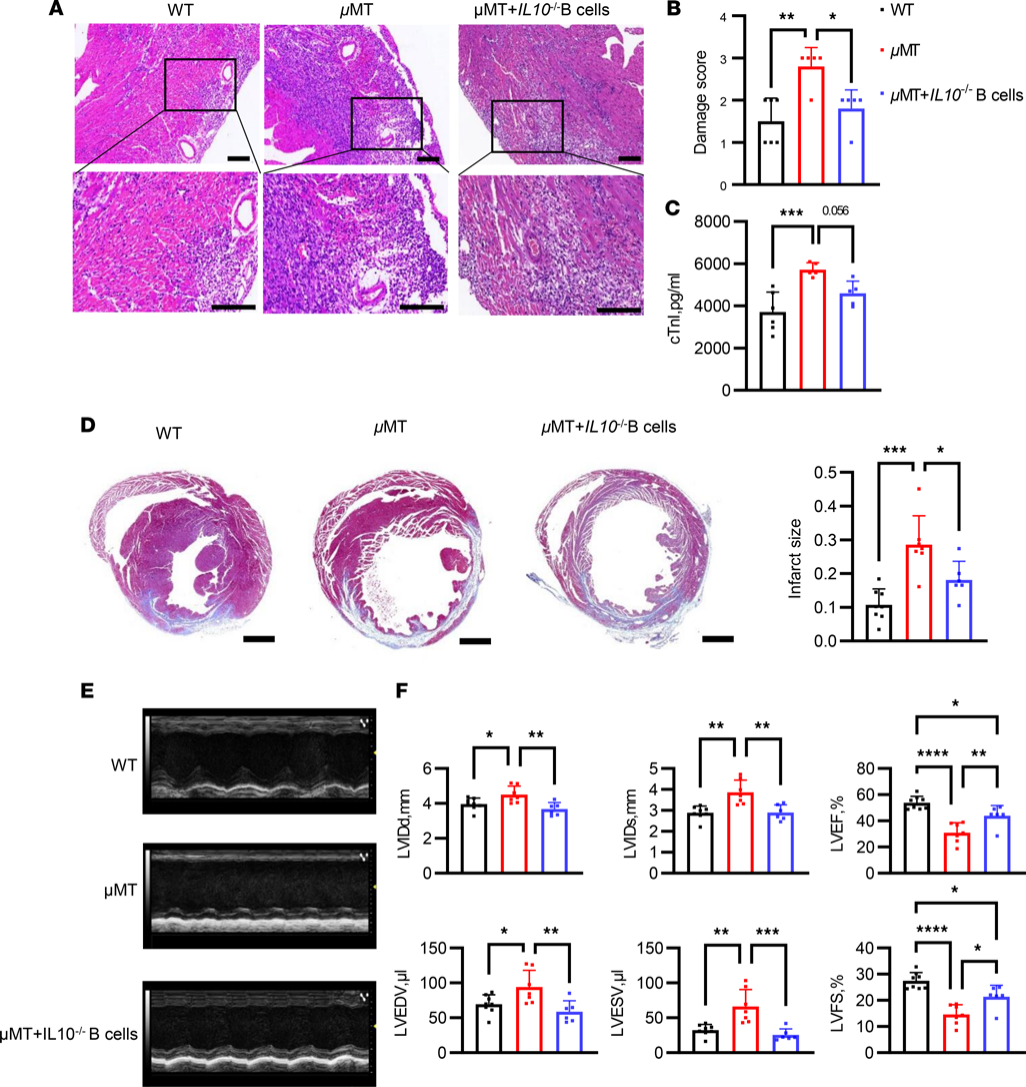
B cells attenuated MIRI via an IL-10–independent mechanism. A total of 1 × 10^7^ splenic B cells from *Il10^–/–^* mice were transferred into μMT mice before and 2 days after myocardial I/R. WT, μMT and, μMT mice transferred with *Il10^–/–^* B cells were subjected to myocardial I/R. (**A**) Representative images of H&E staining of heart slice in each group 3 days after myocardial I/R. Scale bars: 100 μm. (**B**) Damage scores to quantify the extent of tissue injury in each group 3 days after myocardial I/R. (**C**) Serum cardiac troponin I level in each group. (**D**) Representative Masson’s trichrome staining of heart slice and quantification of infarct size after myocardial I/R in each group. Scale bars: 1000 μm. The infarct size was determined by calculating the ratio of the collagen area to the left ventricular area. (**E**) Representative M-mode echocardiography of each group 14 days after I/R. (**F**) Measurements of cardiac function and cardiac size in each group 14 days after I/R. Data are presented as mean ± SEM. *P < 0.05; **P < 0.01; ***P < 0.001; ****P < 0.0001 by 1-way ANOVA test followed by Tukey’s post hoc test (*n* = 6–8 mice per group). cTnI, cadiac troponin I; I/R, ischemia and reperfusion; LVIDd, left ventricular diastolic dimension; LVIDs, left ventricular systolic dimension; LVEF, left ventricular ejection fraction; LVFS, left ventricular fractional shortening.

It has been widely reported that IL-10 produced by B cells is crucial for mitigating tissue injury, including permanent MI (7). Therefore, we next used *Il10^–/–^* mice to study whether IL-10 was the only factor influencing the degree of MIRI. Like the earlier findings with B cell–depleted mice, μMT (B cell–deficient) mice exhibited a significant exacerbation of MIRI. Notably, the adoptive transfer of *Il10*^–/–^ B cells into μMT mice resulted in a reduction in MIRI, evidenced by the decreased serum cardiac biomarkers, myocardial damage score, infarct size, and improved cardiac functions (Supplemental Figure 4 and Figure 3, A–F). In addition, we employed a bone marrow–chimeric system wherein IL-10 deficiency was specifically confined to B cells to assess the impact of *Il10*^–/–^ B cells on MIRI. CD45.1^+^ recipients were lethally irradiated before being reconstituted with 80% μMT bone marrow supplemented with 20% bone marrow from either *Il10^+/+^* or *Il10^–/–^* mice. As a control, we generated bone marrow–chimeric mice with 100% of bone marrow from μMT mice (Supplemental Figure 5). The chimeric mice underwent myocardial I/R 2 months after reconstitution. Histological analysis and echocardiography revealed that mice with *Il10^+/+^* and *Il10^–/–^* bone marrow exhibited attenuated myocardial injury and improved cardiac function in comparison with those reconstituted with 100% μMT bone marrow (Supplemental Figure 6). These findings suggest that B cells play a protective role against MIRI, and the protective effects of B cells involve an IL-10–independent mechanism.

### B cell depletion boosts inflammation of the heart after MIRI.

**Figure 4:**
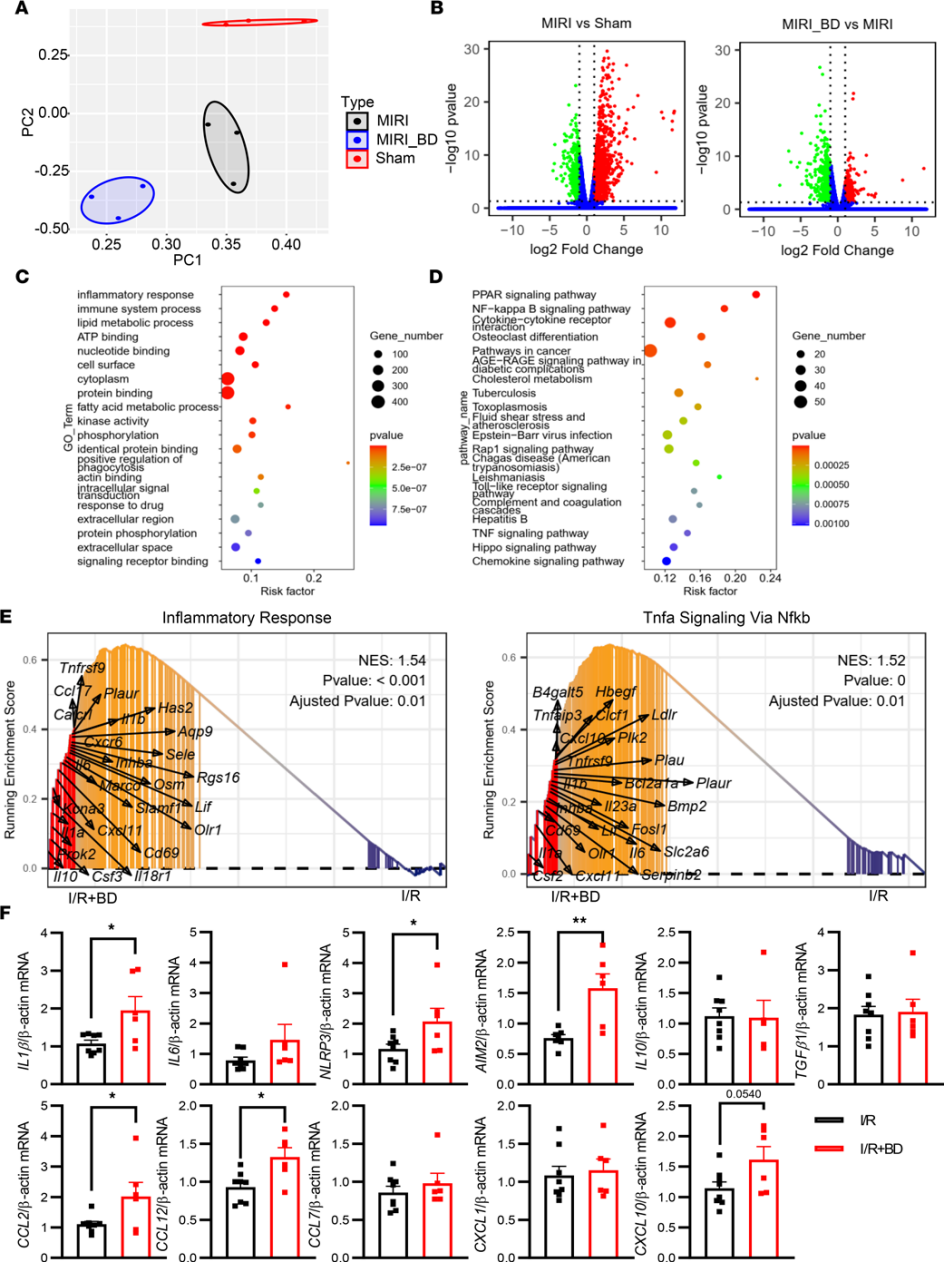
Selective B cell depletion boosted inflammation of the heart 24 hours after myocardial I/R or sham operation. Mice were injected i.v. with 200 μg anti-CD20 to deplete B cells in mice. RNA-seq of myocardial samples from mice with or without B cell depletion (BD) after MIRI or sham operation were detected (*n* = 3 mice per group). (**A**) PCA of RNA-seq expression data among the 3 groups. (**B**) Volcano plots of gene expression profile data in samples of the 3 groups. (**C**) Top 20 GO and (**D**) KEGG enrichment analyses of heart DEGs between mice with or without B cell depletion after MIRI. The *y* axis represents different pathways and the *x* axis represents the percentage of each functional group gene among the total genes. (**E**) In the Hallmark gene sets, GSEA plots with the top 25 leading targets revealed that “inflammatory response” and “TNF signaling via NF-κB” pathways were upregulated in the I/R + BD group compared with the I/R group. (**F**) The mRNA expression levels of inflammatory factors (*Il1b*, *Il6*, *Nlrp3*, *Aim2*, *Il10*, *Tgfb1*, *Ccl2*, *Ccl12*, *Ccl7*, *Cxcl1*, and *Cxcl10*) were quantified from heart tissue of B cell–depleted and control mice 1 day after MIRI. Expression was normalized to that of *Actb* (β-actin). Data are presented as mean ± SEM. *P < 0.05, **P < 0.01 by unpaired, 2-tailed *t* test. MIRI, myocardial ischemia and reperfusion injury; PC, principal component.

As previously mentioned, the increased number of apoptotic cardiac cells after myocardial I/R was observed in mice with B cell depletion or B cell deficiency. Initially, we asked whether B cells could improve cardiomyocyte survival under hypoxia and reoxygenation (H/R) conditions. An annexin V–directed apoptosis assay, however, revealed that B cells failed to directly increase the number of viable cardiomyocytes undergoing H/R in vitro (Supplemental Figure 7). To systematically investigate the mechanisms underlying the protective role of B cells after MIRI, we conducted RNA-seq analysis of mouse hearts. This approach provided insights into global gene expression profiles across 3 groups: Sham, MIRI with B cell depletion, and MIRI without B cell depletion. Principal Component Analysis (PCA) of RNA-seq data revealed distinct clustering of samples into 3 categories, suggesting that gene expression patterns among the Sham, MIRI with B cell depletion, and MIRI without B cell depletion groups were significantly different (Figure 4A). The volcano plots show many differentially expressed genes (DEGs) within the 3 groups (Figure 4B). The heatmap data further demonstrate that the top 100 DEGs were significantly capable of distinguishing among the 3 groups (Supplemental Figure 8). Subsequently, we performed Gene Ontology (GO) and Kyoto Encyclopedia of Genes and Genomes (KEGG) enrichment analyses. In comparison with the sham group, the enriched pathways in the MIRI group included “TNF signaling pathway,” “chemokine signaling pathways,” “NF-κB signaling pathway,” “neutrophil chemotaxis,” and other inflammation-related pathways (Supplemental Figure 9). Interestingly, DEGs between the MIRI and MIRI with B cell depletion groups were also primarily associated with inflammation-related pathways, such as “inflammatory response,” NF-κB signaling pathway,” “cytokine-cytokine receptor interaction,” “TLR signaling pathway,” “TNF signaling pathway,” and “chemokine signaling pathways” (Figure 4, C and D). To more thoroughly understand the mechanism underlying the effect of B cell depletion on MIRI, gene set enrichment analysis (GSEA) was utilized. The analysis identified that the upregulated gene sets in B cell depletion groups were associated with “inflammatory response” and “TNF-α signaling via NF-κB,” suggesting that B cell depletion boosted inflammation (Figure 4E). To further corroborate this, quantitative PCR (qPCR) of the ischemic myocardium was performed. The qPCR results demonstrated that the transcript levels of proinflammatory factors in ischemic myocardium, such as *Il1b*, *Nlrp3*, *Aim2*, *Ccl2*, *Ccl12*, and *Cxcl10*, were significantly elevated in B cell–depleted mice compared with control mice (Figure 4F). Collectively, these findings suggest that selective B cell depletion boosts the inflammatory response.

### Selective depletion of B cells or B cell deficiency delayed the transition from proinflammatory to antiinflammatory phase after myocardial I/R.

**Figure 5:**
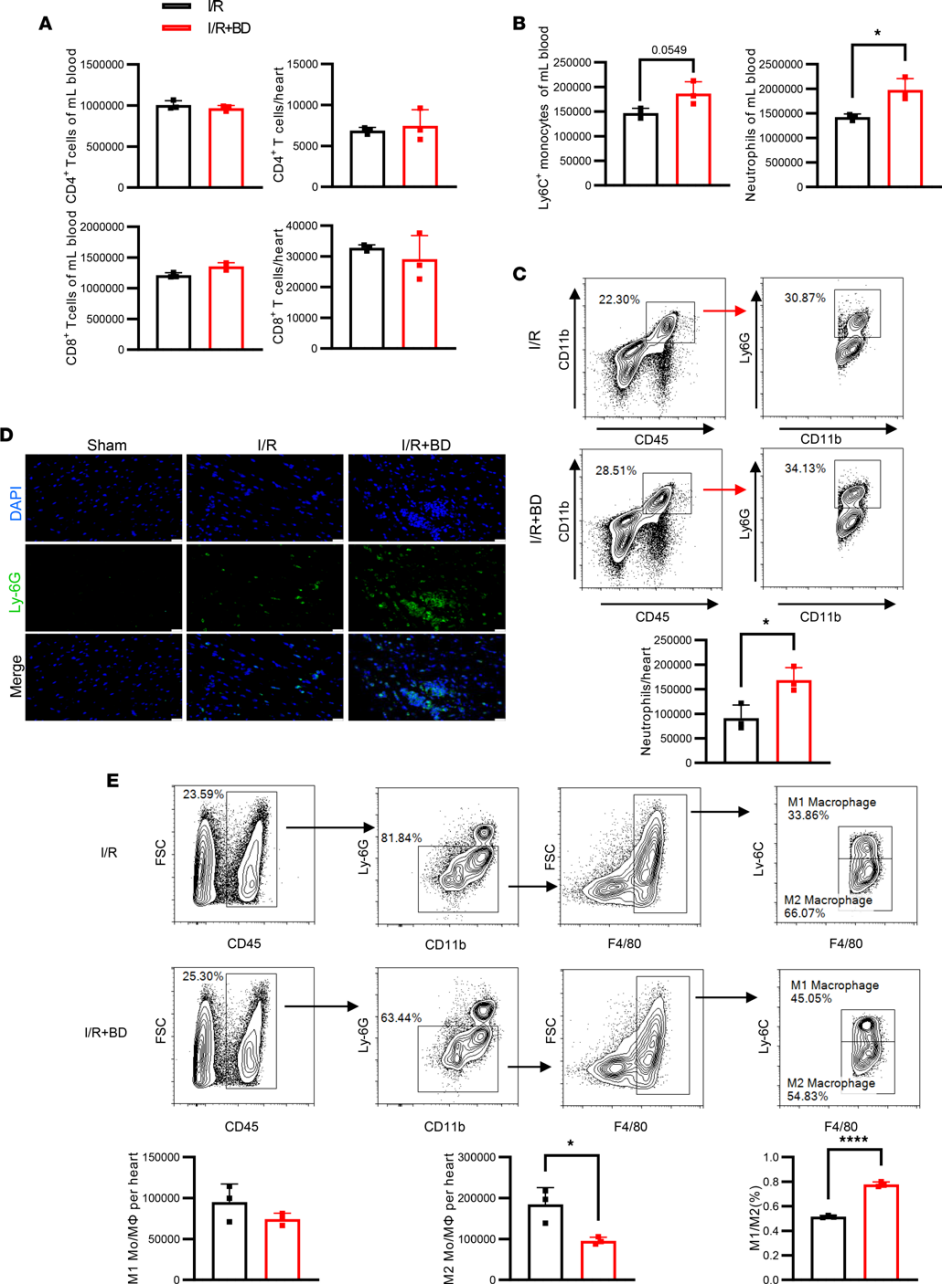
Selective B cell depletion delayed acute inflammation resolution after MIRI. Mice with or without B cell depletion underwent myocardial I/R, and the hearts were harvested and digested 3 days after MIRI. (**A**) Flow cytometry was used to determine the levels of CD4^+^ and CD8^+^ T cells in peripheral blood and heart. (**B**) Ly6C^+^ monocytes and neutrophils in peripheral blood, and (**C**) neutrophils in heart tissue from B cell–depleted and control mice 3 days after MIRI. (**D**) Representative images of immunofluorescence of Ly6G^+^ neutrophils in heart sections from sham-operated, B cell–depleted, and control mice 3 days after MIRI (*n* = 6 mice per group). Scale bars: 50 μm. (**E**) Gating strategy and flow cytometry–based quantification of the number and ratio of CD45^+^CD11b^+^Ly6G^–^F4/80^+^Ly6C^hi^ and CD45^+^CD11b^+^Ly6G^–^F4/80^+^Ly6C^lo^ monocytes/macrophages (Mo/MΦ) on day 3 following I/R in control and B cell–depleted mice. The experiments were independently replicated twice. Data are presented as mean ± SEM. *P < 0.05, ****P < 0.001 by unpaired, 2-tailed *t* test. BD, B cell depletion; I/R, ischemia and reperfusion.

**Figure 6:**
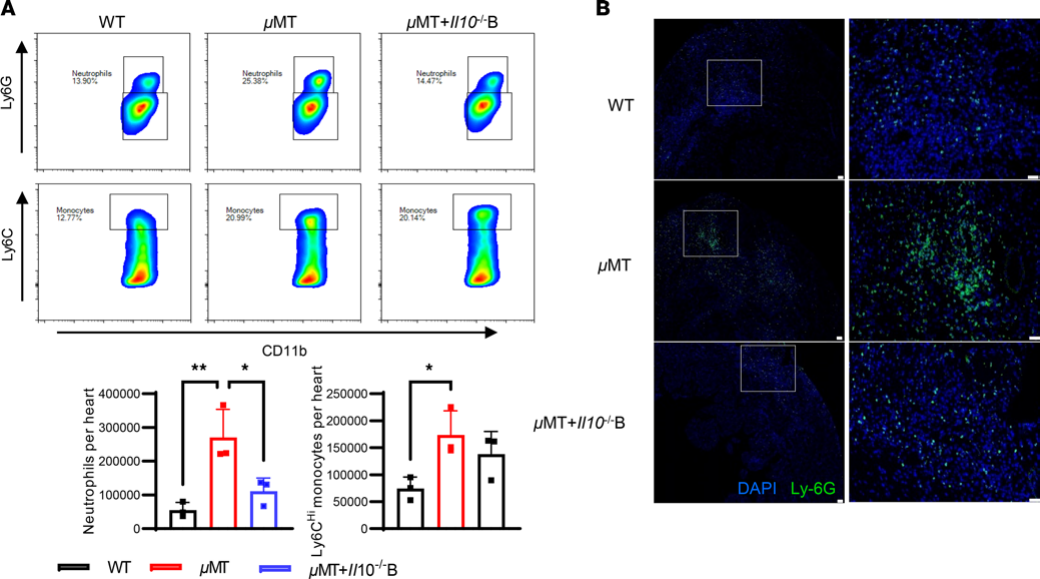
B cell deficiency delayed acute inflammation resolution after MIRI. (**A**) Representative images of flow cytometric analysis and flow cytometry–based quantification of the number of Ly6G^+^ neutrophils and Ly6C^hi^ monocytes in the digested heart from WT mice, μMT mice, and μMT mice adoptively transferred with *Il10^–/–^* B cells 3 days after myocardial I/R, respectively. Data are presented as mean ± SEM. *P < 0.05; **P < 0.01 by 1-way ANOVA test followed by Tukey’s post hoc test (*n* = 3 mice per group). (**B**) Representative images of Ly6G immunofluorescence in heart sections (*n* = 6 mice per group). Scale bars: 50 μm.

Next, we sought to address the mechanisms of the immunoregulatory role of B cells after myocardial I/R. Prior research has reported that Bregs regulate the immune system by modulating the proliferation, differentiation, and death of target immune cells (14). Therefore, we assessed the alterations in immune cells commonly implicated in the pathogenesis of MIRI in mice with or without B cell depletion. The interplay between Bregs and T cells has been discussed in several studies (15). We firstly investigated the levels of T cells after B cell depletion in post-MIRI mice. No substantial differences were observed in the numbers of heart and circulating CD4^+^ T cells or CD8^+^ T cells between mice with and without B cell depletion (Figure 5A). Subsequently, we observed an increasing trend in circulating neutrophils and Ly6C^hi^ monocytes in the absence of B cells after myocardial I/R (Figure 5B). Additionally, we examined the numbers of inflammatory cells in heart tissues. Both flow cytometric analysis of digested hearts and immunohistochemical staining of heart slices revealed more Ly6G^+^ neutrophils 3 days after MIRI in hearts of mice with B cell depletion (Figure 5, C and D). Moreover, 2 monocyte/macrophage subsets (Ly6C^+^ and Ly6C^–^) have been implicated in the transition of inflammatory state after myocardial ischemia (16, 17). Thus, we explored the role of monocytes/macrophages in cardiac repair after myocardial I/R. Notably, although the absolute number of CD45^+^CD11b^+^Ly6G^–^F4/80^+^Ly6C^+^ monocytes/macrophages was not affected regardless of whether B cells were depleted, the total number of Ly6C^–^ monocytes/macrophages was significantly reduced in B cell–depleted mice (Figure 5E). The ratio of Ly6C^+^/Ly6C^–^ monocytes/macrophages was significantly increased in B cell–depleted mice. Moreover, our findings indicated that mice with B cell depletion exhibited an early-phase heightened inflammatory response, evidenced by an increased number of neutrophils and reduced numbers of M2-like macrophages in post-24-hour myocardial tissues compared with those without B cell depletion (Supplemental Figure 10). To further demonstrate whether B cells regulate the transition from inflammatory to reparative phase following MIRI, we analyzed μMT mice. The findings indicated that μMT mice exhibited an increased number of Ly6G^+^ neutrophils and proinflammatory monocytes in the heart, while the adoptive transfer of *Il10^–/–^* B cells into μMT mice resulted in a significant decrease in neutrophils (Figure 6, A and B). The chimeric mouse model also demonstrated a significant reduction in neutrophil infiltration following myocardial I/R in CD45.1 mice reconstituted with a mixed bone marrow composition (80% from μMT mice and 20% from either *Il10^+/+^* or *Il10^–/–^* mice), in contrast with chimeric mice with 100% μMT bone marrow reconstitution (Supplemental Figure 11). Taken together, our results show that unfavorable repair in the absence of B cells might be a consequence of a delayed transition from proinflammation to the reparative phase after myocardial I/R.

### B cells can induce neutrophil apoptosis by contact-dependent interaction and indirectly promote reparative macrophage polarization in vitro and in vivo.

**Figure 7:**
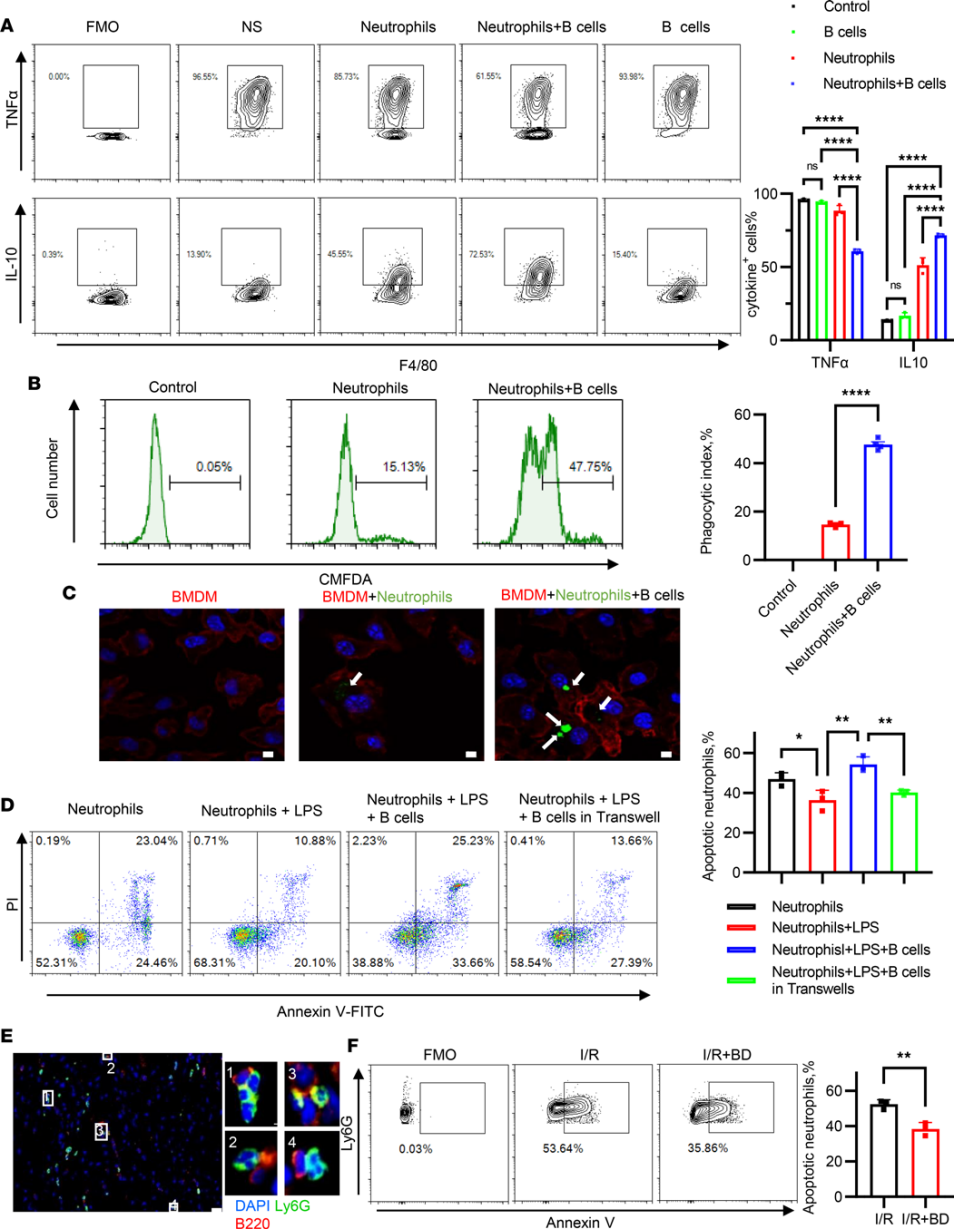
B cells promote macrophage polarization in the presence of neutrophils and induced neutrophil apoptosis via cell-cell interaction. (**A**) Bone marrow–derived macrophages (BMDMs) were incubated with B cells and/or neutrophils, or alone in the presence of 100 ng/mL LPS for 12 hours and 1 μg/mL monensin, a Golgi blocker, for the last 4 hours. Intracellular IL-10 and TNF-α of F4/80^+^ macrophages were detected by flow cytometry. NS, normal saline. (**B**) Freshly isolated neutrophils were labeled with CMFDA and cultured with B cells or alone for 12 hours. Efferocytosis assay was conducted by adding CMFDA-labeled neutrophils cocultured with or without B cells to BMDMs for 60 minutes, as described in the Methods. The percentage of BMDMs phagocytosing CMFDA-labeled neutrophils was quantified using flow cytometric analysis. (**C**) Confocal microscopy images show Alexa Fluor 563–conjugated phalloidin–labeled macrophages phagocytosing CMFDA-labeled neutrophils (*n* = 4 per group). Scale bars: 5 μm. (**D**) Freshly isolated neutrophils were cocultured for 12 hours with B cells or alone. In 1 group, B cells were also cocultured in Transwells. During coculture, cells were treated with 100 ng/mL LPS or vehicle. The percentage of apoptotic CD19^–^Ly6G^+^ neutrophils was determined by flow cytometry. (**E**) Representative images of immunofluorescent staining show the interaction between Ly6G^+^ neutrophils and CD19^+^ B cells in heart sections of mice 3 days after myocardial I/R (*n* = 5). Scale bar: 50 μm. (**F**) The neutrophils from the heart of B cell–depleted and control mice subjected to myocardial I/R were analyzed for annexin V and PI staining. The percentage of annexin V^+^ neutrophils was quantified. The experiments were independently replicated twice. Data are presented as mean ± SEM. *P < 0.05; **P < 0.01; ****P < 0.0001 by 1-way ANOVA test followed by Tukey’s post hoc test (**A**, **B**, and **D**); differences between 2 groups were compared by unpaired, 2-tailed *t* test (**F**). BD, B cell depletion; FMO, fluorescence minus one; I/R, ischemia and reperfusion.

We then investigated the mechanism by which B cells regulate inflammatory responses and protect the heart from MIRI. The transition of monocytes/macrophages from an inflammatory to a reparative subtype marks the resolution of the inflammatory response, which can reduce MIRI. Thus, we examined the association of B cells with macrophage polarization via in vitro coculture system of B cells and macrophages. Lipopolysaccharide (LPS), an inflammatory stimulus, was used to induce M1 polarization of bone marrow–derived macrophages (BMDMs), simulating the inflammatory status in vitro. We observed that B cells failed to directly induce BMDMs toward M2 polarization when cocultured with BMDMs (Figure 7A). Considering that B cell deficiency also altered the levels of circulating and cardiac neutrophils after myocardial I/R, we further asked whether neutrophils were involved in the role of B cells in regulating inflammation. The results showed that B cells downregulated TNF-α levels and upregulated IL-10 levels of BMDMs only in the presence of neutrophils (Figure 7A and Supplemental Figure 12). This implies that neutrophils may be required for B cell–induced macrophage polarization toward the M2 phenotype.

Neutrophils are short-lived cells that rapidly undergo death, primarily through apoptosis. Externalized phosphatidylserine by apoptotic neutrophils can trigger macrophage phagocytosis and subsequently induce M2 macrophage polarization (2). Thus, we speculated that B cells can enhance the phagocytosis of apoptotic neutrophils by macrophages. To test this hypothesis, an in vitro phagocytosis experiment was performed. CMFDA-labeled freshly isolated neutrophils were cultured with or without B cells for 12 hours, and then they were incubated with BMDMs for 1 hour. After quenching nonphagocytosed cells, the macrophages phagocytosing CMFDA-labeled neutrophils were analyzed by flow cytometry and visualized by confocal microscopy. As shown in Figure 7, B and C, the neutrophils that were preincubated with B cells were more readily phagocytosed by BMDMs. Next, we investigated the effect of B cells on neutrophil apoptosis. We observed that LPS treatment significantly decreased the percentage of annexin V^+^ neutrophils compared with control, consistent with previous findings that TLR4 agonists prolonged neutrophil survival (18). Interestingly, apoptotic neutrophils significantly increased after coculture with B cells upon LPS stimulation, whereas B cells in Transwell inserts did not affect neutrophil apoptosis (Figure 7D). Treatment with zVAD-FMK, a specific inhibitor of apoptotic cell death, blocked the effect of B cells on neutrophil death (Supplemental Figure 13). These data suggested that B cells could induce neutrophil apoptosis through cell contact–dependent interactions and indirectly promote macrophage M2 polarization by facilitating neutrophils to be phagocytosed by macrophages in vitro.

We also confirmed these observations in vivo. Immunofluorescent staining demonstrated that most B220^+^ B cells were in close proximity to Ly6G^+^ neutrophils in the ischemic area (Figure 7E). Flow cytometric analyses of digested heart tissues revealed that B cell depletion reduced the percentage of apoptotic neutrophils (Ly6G^+^annexin V^+^) after myocardial I/R (Figure 7F), indicating that the effect of B cells on neutrophil apoptosis can be recapitulated in the in vivo MIRI model.

### The regulatory effects of B cells disappeared after MIRI in the absence of neutrophils.

**Figure 8:**
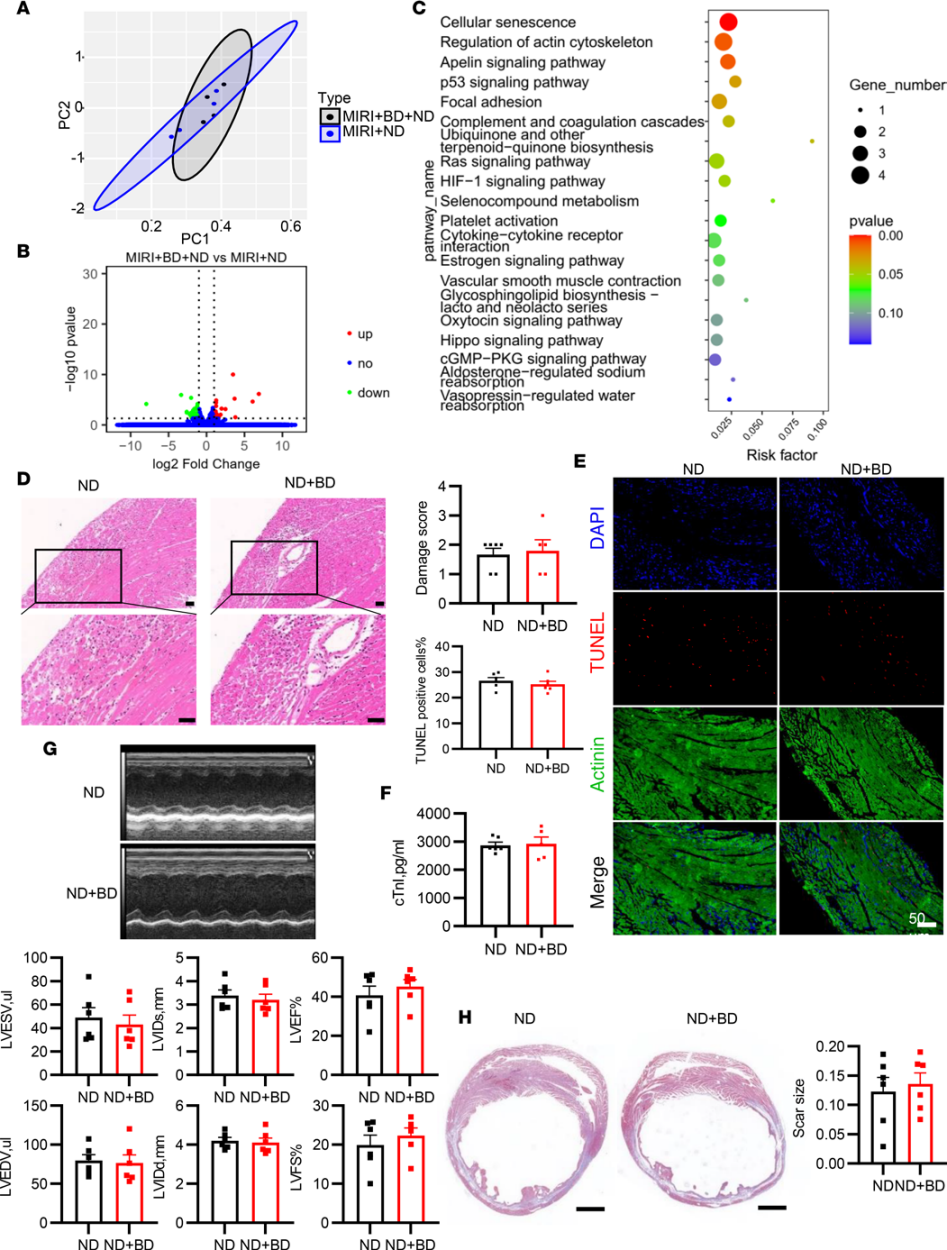
The protective effect of B cells against MIRI vanished in the absence of neutrophils. Neutrophil depletion (ND) was performed by i.p. injection of mAb clone 1A8 (200 μg) once every 3 days. In the condition of ND, mice with or without B cell depletion underwent MIRI. (**A**) Principal component analysis of RNA-seq profile of ischemic myocardium in the 2 groups (*n* = 4 mice per group). (**B**) Volcano plots of gene expression profile data in samples of the 2 groups. (**C**) KEGG enrichment analysis of DEGs between the 2 groups. (**D**) Representative images of H&E staining of heart sections from the 2 groups. Scale bars: 100 μm. (**E**) Apoptotic cardiac cells were detected via TUNEL staining 1 day after myocardial I/R. Scale bar: 50 μm. (**F**) Serum cardiac troponin I level in the 2 groups 1 day after myocardial I/R (*n* = 5–6 mice per group). (**G**) Echocardiographic measurements of cardiac function and cardiac size between B cell–depleted mice and controls 14 days after MIRI in the condition of ND. (**H**) Representative images of Masson’s trichrome staining of heart slice and quantification of infarct size after myocardial I/R in each group. Scale bars: 1000 μm. The infarct size was determined by calculating the ratio of the collagen area to the left ventricular area. BD, B cell depletion; cTnI, cardiac troponin I; I/R, ischemia and reperfusion; LVIDd, left ventricular diastolic dimension; LVIDs, left ventricular systolic dimension; LVEF, left ventricular ejection fraction; LVFS, left ventricular fractional shortening; TUNEL, terminal deoxynucleotidyl transferase–mediated nick-end labeling. Data are presented as mean ± SEM. Differences between the 2 groups were compared by unpaired, 2-tailed *t* test.

To further confirm whether the regulatory effect of B cells is dependent on neutrophils, we repeated the investigation of the immunoregulatory role of B cells in MIRI after predepleting neutrophils. Treatment with anti-Ly6G antibody resulted in a substantial reduction in circulating neutrophils without affecting B cell levels (Supplemental Figure 14). In contrast to the findings above, when co-depleting neutrophils, PCA of RNA-seq data from the ischemic myocardium revealed no distinct clustering between groups with or without B cell depletion (Figure 8A). Moreover, B cell depletion resulted in significantly fewer DEGs, among which KEGG enrichment analysis did not include inflammation-related pathways (Figure 8, B and C). We also assessed the effect of B cells on cardiac function and tissue injury following neutrophil depletion. Mice subjected to co-depletion of neutrophils and B cells did not exhibit worsened tissue injury, as indicated by cardiac function, tissue damage score, infarct size, cTnI levels, and TUNEL^+^ cardiac cells, in comparison to mice undergoing only neutrophil depletion (Figure 8, D–H). This evidence suggested that the immunoregulatory effect of B cells in MIRI is attenuated in the absence of neutrophils.

### The identification of the potential molecule involved in B cell–induced neutrophil apoptosis.

**Figure 9:**
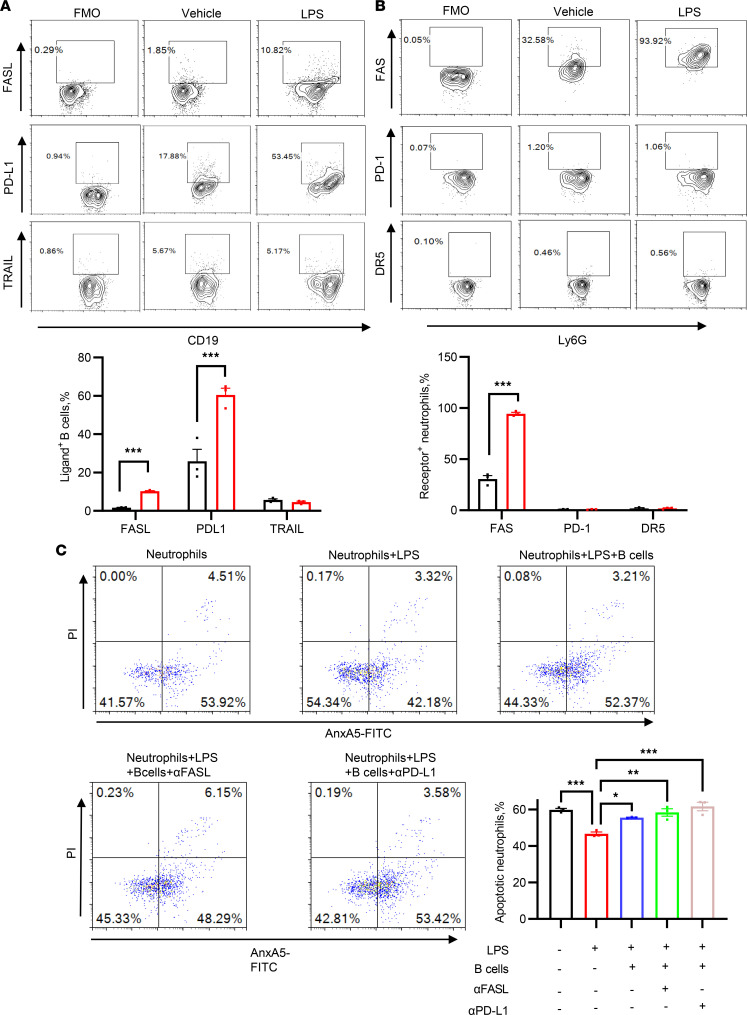
The classical death ligand–receptor pairs did not mediate the proapoptotic role of B cells against neutrophils. (**A**) The expression of FASL, PD-L1, and TRAIL on B cells with or without LPS (100 ng/mL) treatment. (**B**) The expression of FAS, PD-1, and DR5 on neutrophils. (**C**) Representative flow cytometry images for the detection and quantification of apoptotic neutrophils in each group. In the presence of LPS (100 ng/mL) or vehicle, freshly isolated neutrophils were cocultured for 12 hours with B cells that were preincubated with anti-FASL (10 μg/mL), anti–PD-L1 (10 μg/mL), or vehicle. The apoptotic neutrophils were detected using an annexin V/PI assay. Data are presented as mean ± SEM. *P < 0.05; **P <0.01; ***P < 0.001 by unpaired, 2-tailed *t* test (**A** and **B**) or 1-way ANOVA test followed by Tukey’s post hoc test (**C**). The experiments were independently replicated twice. FMO, fluorescence minus one; αFASL, anti-FASL antibody; αPD-L1, anti–PD-L1 antibody.

As mentioned earlier, B cells induce neutrophil apoptosis through direct cell-cell contact. Subsequently, we sought to identify the molecules mediating the interaction between B cells and neutrophils, potentially leading to neutrophil apoptosis. Previous studies have reported that B cells can induce apoptosis of other immune cells through various death-related ligand-receptor pairs, such as FASL-FAS, PD-L1–PD-1, and TRAIL-DR5 (19, 20). Initially, we examined the expression of these ligand-receptor pairs on B cells and neutrophils, respectively. Isolated splenic B cells were found to constitutively express low levels of FASL and TRAIL (<5%) and modest levels of PD-L1 (17.88%) under steady-state conditions. An inflammatory stimulus significantly increased the expression of PD-L1, but not FASL or TRAIL, on B cells (Figure 9A). Conversely, bone marrow neutrophils expressed a certain level of FAS but very low levels of PD-1 and DR5 (<2%) under steady-state conditions. LPS treatment further increased the expression of FAS, but did not influence the levels of PD-1 or DR5 on neutrophils (Figure 9B). In vivo detection revealed similar changes in these ligand-receptor pairs on B cells and neutrophils (Supplemental Figure 15). The low expression levels of TRAIL on B cells and DR5 on neutrophils indicated that the TRAIL/DR5 axis is unlikely to mediate neutrophil apoptosis. We next explored whether the PD-L1/PD-1 or FASL/FAS axis could be implicated in B cell–induced neutrophil apoptosis. However, neutrophil apoptosis induced by B cells remained unaffected when the coculture was performed in the presence of anti-FASL or anti–PD-L1 blocking antibodies (Figure 9C), suggesting traditional death ligands (i.e., FASL and PD-L1) were not responsible for the proapoptotic effect of B cells on neutrophils.

**Figure 10:**
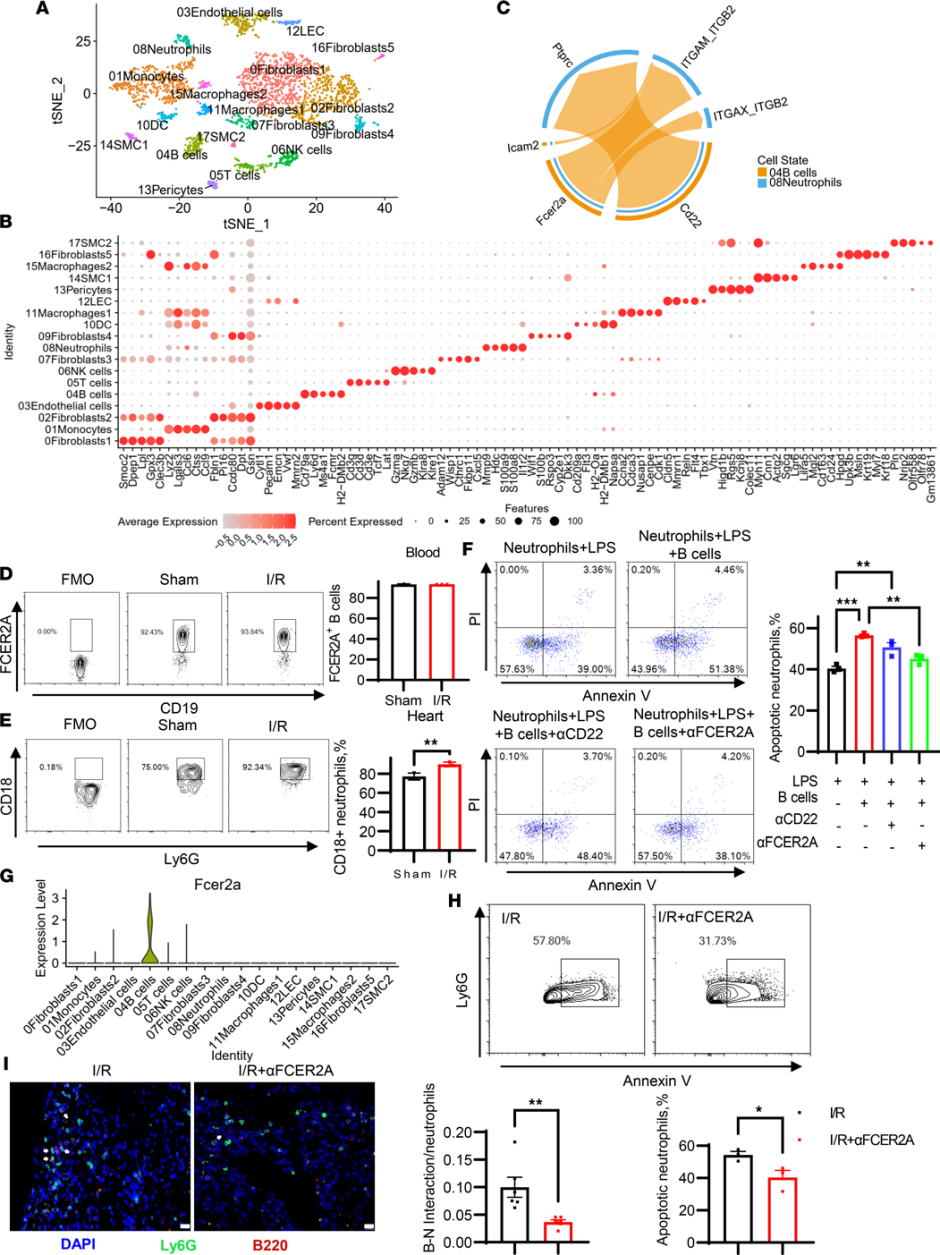
The identification of the molecule on B cells that induced neutrophil apoptosis. (**A**) tSNE plot from the pooled single-cell RNA sequencing of noncardiomyocyte cells from hearts at 1 and 3 days after myocardial ischemia. (**B**) Dot plot visualization of the top 5 different marker genes in each cluster. Dot size represents the percentage of expression per cluster; color gradient represents average expression levels per cell. (**C**) The chord diagram shows the significant inferred interaction from the source cell type (i.e., B cells) with the target cell type (i.e., neutrophils). The arrow thickness is proportional to the calculated communication probability. (**D**) FCER2A expression levels on circulating B cells of sham-operated or post-I/R mice. (**E**) CD18 expression levels on neutrophils in heart of sham-operated or post-I/R mice. (**F**) Representative flow cytometry images for the detection and quantification of apoptotic neutrophils in each group. In the presence of LPS (100 ng/mL), freshly isolated neutrophils were cultured for 12 hours with or without B cells that were preincubated with anti-CD22 (10 μg/mL), anti-FCER2A (10 μg/mL), or vehicle. (**G**) The violin plot shows *Fcer2a* expression levels in each cluster after myocardial ischemia. (**H**) Mice were injected with anti-FCER2A antibody (150 μg per mouse, i.p.) or isotype immediately after I/R. The apoptotic neutrophils from hearts were detected by flow cytometry 3 days after myocardial ischemia. The percentage of annexin V^+^ neutrophils was quantified. (**I**) Representative images of immunofluorescent staining show the interaction between Ly6G^+^ neutrophils and CD19^+^ B cells in heart sections of mice 3 days after MIRI. The experiments were independently replicated twice. Scale bars: 50 μm. Data are presented as mean ± SEM. *P < 0.05, **P < 0.01, ***P < 0.001 by 1-way ANOVA test followed by Tukey’s post hoc test (**D**, **E**, **I**, and **H**); differences between 2 groups were compared by unpaired, 2-tailed *t* test (**F**). αFCER2A, anti-FCER2A antibody; FMO, fluorescence minus one; I/R, ischemia and reperfusion.

**Figure 11:**
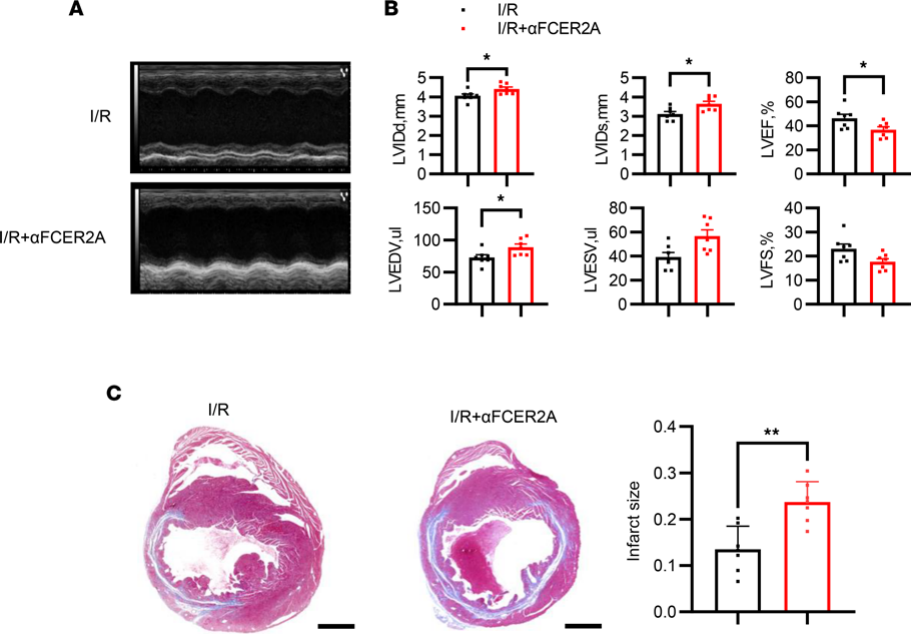
Exacerbation of MIRI by in vivo FCER2A blockade. Mice were injected with anti-FCER2A antibody (150 μg per mouse, i.p.) or isotype immediately after I/R. (**A**) Representative M-mode echocardiography images of each group 14 days after I/R. (**B**) Measurements of cardiac function and size in each group 14 days after I/R. (**C**) Representative Masson’s trichrome staining of heart slices and quantification of infarct size after myocardial I/R in each group. Scale bars: 1000 μm. The infarct size was determined by calculating the ratio of the collagen area to the left ventricular area. Data are presented as mean ± SEM. *P < 0.05, **P < 0.01 by unpaired *t* test. αFCER2A, anti-FCER2A antibody; I/R, ischemia and reperfusion; LVIDd, left ventricular diastolic dimension; LVIDs, left ventricular systolic dimension; LVEF, left ventricular ejection fraction; LVFS, left ventricular fractional shortening; I/R, ischemia and reperfusion.

To delve deeper into the molecules mediating the interaction between B cells and neutrophils, we conducted single-cell RNA-seq (scRNA-seq) analysis on the noncardiomyocyte fraction of murine hearts on days 1 and 3 following myocardial ischemia, as previously described (21). Unbiased clustering identified 18 cell clusters, including 1 cluster of B cells (marked by *Cd79a*, *Ly6d*, *Ms4a1*, and *Fcmr*) and 1 neutrophil cluster (marked by *Mmp9*, *Hdc*, and *S100a9*) (Figure 10, A and B). Utilizing a data set of ligand-receptor pairs, our analysis indicated that *Fcer2a*-*Itgam*/*Itgb2*, *Fcer2a*-*Itgax*/*Itgb2*, and *Cd22*-*Ptprc* may mediate the interaction between B cells and neutrophils (Figure 10C). ITGB2 is known to form various integrin heterodimers by pairing with multiple α chains, including ITGAM and ITGAX. It has been established that cross-linking integrins on neutrophils can expedite their apoptosis (22, 23). We hypothesized that FCER2A on B cells might induce neutrophil apoptosis through an interaction involving FCER2A and integrins. We then analyzed the gene expression distribution of signaling genes associated with these ligand-receptor pairs. Unfortunately, we could not evaluate FCER2A expression on cardiac B cells due to the removal of FCER2A from the cell surface during collagenase digestion of the heart (24). However, our analysis revealed that nearly 90% of circulating B cells expressed FCER2A (Figure 10D). In addition, ITGB2 was expressed on the majority (>90%) of neutrophils (Figure 10E). To validate the insights gained from scRNA-seq data, B cells were preincubated with either anti-CD22 or anti-FCER2A blocking antibodies and subsequently cocultured with neutrophils. Apoptotic assays demonstrated that B cells blocked with anti-FCER2A, but not anti-CD22, were ineffective in inducing neutrophil apoptosis compared with the control (Figure 10F), suggesting that FCER2A on B cells may play a role in neutrophil apoptosis. We further investigated the impact of FCER2A on neutrophil apoptosis in vivo. The scRNA-seq data revealed specific expression of FCER2A in B cells following myocardial ischemia (Figure 10G). Injection (i.v.) of the anti-FCER2A antibody inhibited neutrophil apoptosis in digested heart tissue after MIRI compared with the control (Figure 10H). Immunohistochemical staining also revealed that administration of the anti-FCER2A antibody reduced the physical interaction between B cells and neutrophils (Figure 10I). We also investigated the impact of FCER2A blockade on in vivo heart function recovery. The blockade of FCER2A exacerbated MIRI, characterized by an exacerbated cardiac remodeling (Figure 11, A and B) and enlarged fibrosis (Figure 11C) area compared with the isotype group. Taken together, this evidence indicates that FCER2A plays a pivotal role in mediating the proapoptotic effect of B cells on neutrophils after MIRI.

## Discussion

Reperfusion injury significantly undermines the advantages of revascularization after MI. While it is established that an exacerbated inflammatory response contributes to secondary damage following myocardial I/R, the precise mechanisms governing the regulation of post-MIRI inflammation remain incompletely understood. Given their role as antigen presenters, B cells are poised to fulfill various functions within inflamed tissues. Recent studies by Luigi Adamo and colleagues have suggested that myocardial B cells play a modulatory role in cardiac immunology, influencing both uninjured and injured murine hearts (25–27). However, the potential involvement of B cells in myocardial I/R has been minimally explored. In the present study, several observations were made regarding MIRI, inflammation resolution, Bregs, and neutrophil apoptosis. Our findings revealed the rapid recruitment of B cells and Bregs within the heart following myocardial I/R. Furthermore, B cell depletion or deficiency resulted in exacerbated myocardial injury and profound alterations in the inflammatory milieu within the ischemic heart. FCER2A^+^ B cells were identified as potential contributors to the resolution of the inflammatory response after MIRI by inducing neutrophil apoptosis in a cell-cell contact manner. These observations highlight the rapid emergence of Bregs after myocardial I/R, playing a crucial role in suppressing MIRI and modulating downstream inflammatory responses.

A previous study reported that B cells produced proinflammatory cytokines that promote the recruitment of proinflammatory myeloid cells in permanent coronary occlusion MI (6). In contrast, Lan Wu et al. found an enrichment of IL-10–producing B cells in cardiac adipose tissues, highlighting their significant contribution to the antiinflammatory process that terminates MI-induced inflammation (7). Moreover, a recent study by Jiao Jiao et al. revealed that adoptive transfer of IL-10–producing B cells limited ventricular remodeling after MI through decreasing enrichment of Ly6C^hi^ monocytes (28). A similar result was observed in the present MIRI model. Our study findings uncover that B cells facilitate the resolution of proinflammatory responses following MIRI in a neutrophil-dependent manner. In addition to our findings, these contrasting roles of B cells have been also observed in other organs. For instance, a previous study demonstrated that peritoneal B cells induce functional renal injury, while nonperitoneal B cells attenuate renal injury after I/R (29). These findings suggested that B cells play a complex role in the development of injury after myocardial I/R, emphasizing the need for a comprehensive understanding of the role of B cells in MIRI to better define B cell subsets as potential targets for therapeutic strategies.

Neutrophils, as the first immune cell type to infiltrate the ischemic myocardium, experience accelerated and accentuated infiltration during reperfusion (17). The programmed death of neutrophils is crucial for suppressing the release of proteolytic enzymes and proinflammatory mediators after MIRI. Neutrophil apoptosis, a hallmark of inflammation resolution, contributes to various mechanisms, including the production of antiinflammatory mediators and the exposure of eat-me signals (2). Macrophages engulfing apoptotic neutrophils activate an antiinflammation response to repair injured tissue. A prior study demonstrated that neutrophil depletion impairs the polarization of macrophages toward a reparative phenotype, worsening cardiac function and increasing cardiac fibrosis in permanent-MI mice (30). In our study, we observed that B cell depletion or deficiency delays the transition from a proinflammatory to a reparative phase following MIRI. Additionally, in vitro experiments indicated that B cells do not directly influence macrophage polarization but instead promote M2-type macrophage differentiation indirectly by accelerating the apoptosis of neutrophils, facilitating their phagocytosis by macrophages. The protective effect of B cells diminishes in the absence of neutrophils. Consistent with our findings, a previous report suggested that genetic depletion of B cells impedes the neutrophil apoptosis process, ultimately leading to pathological fibrotic interstitial lung disease (31). Therefore, the regulation of neutrophil apoptosis is critical for maintaining a dynamic balance between proinflammatory and antiinflammatory responses. Our data suggest that B cells could be a potential regulator of neutrophil apoptosis, and future studies may further elucidate the specific cell populations and factors involved in neutrophil apoptosis following myocardial I/R.

Regulatory cytokines, including IL-10 and TGF-β1, and adenosine produced by B cells, are commonly associated with the repair of acutely injured tissue (29, 32–34). However, the molecular mechanisms governing the inflammatory regulation of B cells after myocardial ischemia remain less understood. IL-10 production has been extensively studied as the mechanism underlying the regulatory function of B cells, playing a crucial role in Breg-mediated protection against MI (7, 28). Despite IL-10 being considered a hallmark of Bregs, IL-10–independent mechanisms have been identified in various inflammatory disease models (35). In this study, we observed that B cell subsets appeared to modulate the inflammatory milieu in myocardial ischemia through an IL-10–independent mechanism involving cell-cell contact. Previous reports suggested that B cells employ suppressive mechanisms via cell surface–expressed death ligands such as FASL and PD-L1 (20, 36). However, our in vitro experiments showed that, although inflammatory stimuli upregulated the expression of FASL and PD-L1 on B cells, blocking these ligands did not influence the effect of B cells on neutrophil apoptosis. This observation aligns with the understanding that Bregs, unlike Tregs, do not represent a distinct lineage and exhibit diverse phenotypes and functional modalities (37). The varied functional capacity likely arises from the differential signaling of B cells in specific inflammatory disease contexts. To delve into the complex interaction between the microenvironment and B cells in the context of myocardial ischemia, we employed scRNA-seq in this study. The data from scRNA-seq unveil a role for B cells in promoting neutrophil apoptosis through the interaction of FCER2A with integrins. FCER2A (CD23) is the low-affinity receptor for immunoglobulin E and is prominently expressed on mature B cells, particularly those of the B-2 subtype. In mice, FCER2A is acknowledged as a surface marker for 2 subsets of Breg cells: transitional 2 marginal zone precursor Bregs and GM-CSF– and IL-15–induced Bregs (3, 38). Hence, the Bregs identified in our study, bearing a resemblance to B-2 cells, diverge from those observed by Lan Wu et al. In Lan Wu et al.’s investigation, the Bregs were primarily located in pericardial adipose tissues, conferring protection against myocardial ischemia mainly through paracrine IL-10 release. These cells exhibited a phenotype closely resembling CD5^+^ B-1a cells. It is important to note that our study revealed a minimal presence of B-1a cells in the ischemic heart, while the predominant B cell subtype in this context was B-2. Although direct evidence of FCER2A-mediated effects on neutrophil apoptosis is lacking, a prior study has indicated that FCER2A could specifically interact with β2 integrin adhesion molecule complexes (39), and cross-linking of β2 integrins potentially induced neutrophil apoptosis (40). These findings support our observations that B cells interacted with neutrophils and induced neutrophil apoptosis via FCER2A-integrin interactions in the context of myocardial ischemia. Nevertheless, further research is warranted to investigate the downstream signaling pathways through which FCER2A-integrin interactions induce neutrophil apoptosis.

The quantity of myocardial B cells is lower than that of neutrophils in the injured heart, suggesting a potentially minor impact of B cells on neutrophil apoptosis in theory. However, it is essential to recognize that the influence of B cells on neutrophil apoptosis, and consequently on the inflammatory response, represents a cascade amplification process. The dynamic interplay between these cell types has the potential to exert a significant influence on the resolution of inflammation, thereby impacting the overall cardiac repair process following myocardial I/R. Notably, recent findings from Luigi Adamo et al. revealed that myocardial B cells are primarily localized in the microvasculature, the route through which neutrophils enter the myocardial tissue (25, 26). This positioning increases the likelihood of contact between myocardial B cells and neutrophils. Moreover, given the observed upregulation of various regulatory molecules, including TGF-β1 and adenosine, produced by B cells after MIRI, along with the increased expression of chemokines such as *Ccl2* (as illustrated in Figure 4F) known to act as a chemoattractant for Ly6C^hi^ monocytes following B cell depletion in the MIRI, further investigations are necessary to delve into additional mechanisms mediating the regulatory functions of B cells in the context of MIRI.

In summary, our study elucidates the recruitment of Bregs to the heart following myocardial I/R, leading to increased expression of immunoregulatory mediators. Depletion or deficiency of B cells exacerbates proinflammatory responses and worsens MIRI. Mechanistically, we found that B cells interact with neutrophils, promoting their apoptosis through FCER2A-integrin interactions, subsequently contributing to M2-type macrophage polarization via facilitating macrophage efferocytosis. Our data suggest that refining therapeutic strategies for MIRI by harnessing Breg populations could be beneficial in controlling inflammation initiation and transition after myocardial I/R.

## Methods

Methods and reagent details not described here can be found in the supplemental material.

### Sex as a biological variable.

In this study, we chose to utilize male mice for our MI model due to the inherent hormonal cycles in female mice, which may introduce variability in B cell function (41). Additionally, this decision is in line with common practice observed in the literature, where early studies for constructing MI models predominantly employed male mice. It is crucial to note that, at present, the transferability of our findings to female mice remains unknown. Future research endeavors specifically designed to investigate potential sex-specific differences are warranted to expand our understanding of the broader implications of our study.

### Mice.

B6.129S2-*Ighm^tm1Cgn^*/J (μMT) and B6(Cg)-*Il10^tm1.1Karp^*/J (Vert-X) mice were obtained from Jackson Laboratory. C57BL/6J, B6/JGpt-Il10^em1Cd4885^/Gpt, and C57BL/6JGpt-Ptprc^em1Cin(p.K302E)^/Gpt mice were purchased from GemPharmatech. Mice were maintained and bred in pathogen-free conditions.

### MIRI model.

Eight- to 12-week-old C57BL/6J male mice were used to construct the MIRI model, as described previously (42). Briefly, the mice were anesthetized with 4% isoflurane, and anesthesia was maintained throughout the experiment with 1%–2% isoflurane. Mice were placed in the right lateral decubitus position. A 0.5 cm oblique incision was made at a site 2 mm away from the left sternal border, and the heart was exposed by making a 6–8 mm incision in the third intercostal space. With the aid of a surgical microscope, the left anterior descending coronary artery was ligated with a 7-0 suture for 1 hour before the release of the ligature. The establishment of I/R was ascertained by regional cyanosis and return of normal reddish color in the infarct areas. All mice after experiments were euthanized by cervical dislocation under anesthesia. All experiments in the study were performed and analyzed in a blinded manner. To determine the appropriate sample sizes for each experiment, we utilized the biomath online tool (http://biomath.info/power/), tailoring calculations to the specific design of each study.

### In vivo B cell depletion and neutrophil depletion.

B cells were depleted via i.v. injection of anti–mouse CD20 antibody (200 μg per mouse) immediately after myocardial I/R. Neutrophil depletion was performed by intraperitoneal (i.p.) injection of mAb clone 1A8 (200 μg per mouse) 2 days before I/R and once every 3 days on the following days. Control mice received corresponding isotype i.v. or i.p. injection. In vivo cell depletion was ascertained by flow cytometry.

### Preparation of single-cell suspensions of heart.

The protocol for preparation of single-cell suspensions from the murine heart was as previously described (43), with modifications. Isolated hearts were extensively flushed with cold PBS and finely minced into small pieces and placed in 3 mL of digestion buffer (450 U/mL collagenase I, 125 U/mL collagenase XI, 60 U/mL hyaluronidase, and 60 U/mL DNase I). The tissue was incubated at 37°C for 15 minutes on a rotatory shaker at 30 rpm. After incubation, a 10-mL serological pipette was used to gently blow and mix the tissue digestion buffer. The suspension was again incubated at 37°C and triturated twice more (45 minutes of total digestion time). All cell suspensions were filtered through a 70-μm cell strainer. Filtered single-cell suspensions were resuspended in a 2% FBS solution and centrifuged at 400*g* for 5 minutes. Cell pellets were resuspended in 30% Percoll and overlaid with PBS. The gradient was centrifuged at 700*g* for 30 minutes without brake at room temperature. The interface containing dead cells and cell debris was removed. The live cells at the bottom of the tube were washed with PBS twice. Red blood cell lysis was performed using Red Blood Lysis Buffer as per the manufacturer’s recommendation (Solarbio Life Science, R1010). Cell pellets were washed twice with PBS.

### Flow cytometry.

Cell suspensions isolated from the heart were analyzed by flow cytometry. Dead cells were stained with Zombie Yellow Fixable Viability Kit for 20 minutes at room temperature, and Fc-block was performed with anti–mouse CD16/CD32 for 10 minutes at 4°C. Subsequently, extracellular staining was performed with an antibody cocktail at 4°C. All antibodies were used at 1:100 dilution. For intracellular cytokine detection, after completing extracellular staining, cells were fixed/permeability by using an intracellular cytokine staining kit (eBiosciences) for 20 minutes as per the manufacturer’s recommendation. The fixed cells were sequentially stained with corresponding cytokine antibodies for 30 minutes at 4°C. Two fluorescence controls were used for the multicolor flow cytometry: fluorescence minus one (FMO) or isotope control. For FMO, an antibody cocktail without the color of interest was used to determine the boundary. For an isotype control, an isotype antibody was used to determine the boundary. Cellular fluorescence was determined on a NovoCyte cytometer and NovaExpress software.

### Induction and quantification of neutrophil apoptosis.

Freshly isolated neutrophils (1 × 10^5^/well) with or without cocultured B cells (4 × 10^5^/well) were treated with LPS (100 ng/mL) or vehicle in a 24-well plate overnight. For Transwell experiments, B cells were firstly loaded in a Transwell (Millipore, 0.4 μm, 4 × 10^5^ cells) and subsequently placed in a 24-well plate with neutrophils (1 × 10^5^/well). In some experiments, B cells were preincubated with anti–PD-L1 (10 μg/mL), anti-FASL (10 μg/mL), anti-TRAIL (10 μg/mL), and anti-FCER2A (10 μg/mL).

To assess neutrophil apoptosis, after incubation overnight, cell suspensions were washed twice with FACS solution, Fc blocked with anti–mouse CD16/CD32 for 10 minutes, and stained with Brilliant Violet 785–labeled anti–mouse Ly6G (1:100) and APC-labeled anti–mouse CD19 (1:100) for 30 minutes at 4°C. The cell suspensions were then washed twice with PBS. FITC-labeled annexin V was then used to assess neutrophil apoptosis using flow cytometry as described by the manufacturer.

### Efferocytosis assays.

Efferocytosis assays were performed as described previously (44), with modifications. Briefly, freshly isolated neutrophils were stained using CMFDA as per the manufacturer’s recommendation (YEASEN Biotechnology, 40721ES50). CMFDA-labeled neutrophils (5 × 10^5^) were incubated with or without B cells (2 × 10^6^) overnight. Then, the CMFDA-labeled neutrophils were cocultured with BMDMs (5 × 10^5^) that were preseeded in a 24-well plate. After a 1-hour incubation, cells were rinsed twice with PBS, and supernatants were removed. BMDMs were collected and extracellular fluorescence associated with membrane-bound but nonengulfed apoptotic cells and bodies were quenched with trypan blue (0.04% in PBS). The proportion of BMDMs that exhibited increased fluorescence (corresponding to phagocytosis of fluorescently labeled apoptotic neutrophils) was determined by flow cytometry. For microscopic observation, after the treatment, BMDMs were fixed, permeabilized, and stained with iFluor 647 phalloidin (YEASEN Biotechnology, 40762ES75). The nuclei were visualized by staining with DAPI. Microscopic analysis was performed on a Leica inverted fluorescence confocal microscope.

### Statistics.

All numerical data are presented as mean ± SEM. Independent replicates for each data point (*n*) are identified in figure legends. Data graphing and statistical analysis were performed using Prism 9. Differences between 2 groups were compared by unpaired, 2-tailed *t* test. Differences between more than 2 groups were assessed by 1-way ANOVA followed by Tukey’s post hoc test. A *P* value of less than 0.05 was regarded as statistically significant.

### Study approval.

All experimental procedures involving animals were approved by the Animal Ethics Committee of West China Hospital, Sichuan University. All animal experiments were performed according to the guidelines from Directive 2010/63/EU of the European Parliament on the protection of animals used for scientific purposes or the NIH *Guide for the Care and Use of Laboratory Animals* (National Academies Press, 2011).

### Data availability.

The data used to generate the figures are concisely compiled in the supplemental Supporting Data Values file. The raw sequence data reported in this paper have been deposited in the Genome Sequence Archive (Genomics, Proteomics & Bioinformatics 2021) in the National Genomics Data Center (Nucleic Acids Res 2022), China National Center for Bioinformation/Beijing Institute of Genomics, Chinese Academy of Sciences (accession: PRJCA022558) that are publicly accessible at https://ngdc.cncb.ac.cn/gsa Moreover, any necessary supporting analytic codes can be provided by the corresponding author upon request.

## Author contributions

FH, LC, and MC contributed to study design and manuscript writing. FH, JZ, HZ, TQ, LC, KJ, and YL contributed to investigation and methodology. YW contributed to material or technique support. FH, YX, and MC contributed to conceptualization and funding acquisition. All authors read and approved the manuscript. The order of co–first author was based on the length of engagement of the individual authors with the project.

## Supplementary Material

Supplemental data

Supporting data values

## Version 1 01/30/2024

In-Press Preview

## Version 2 02/22/2024

Electronic publication

## References

[1] Hausenloy DJ, Yellon DM (2016). Ischaemic conditioning and reperfusion injury. Nat Rev Cardiol.

[2] Prabhu SD, Frangogiannis NG (2016). The biological basis for cardiac repair after myocardial infarction: from inflammation to fibrosis. Circ Res.

[3] Rosser EC, Mauri C (2015). Regulatory B cells: origin, phenotype, and function. Immunity.

[4] Long W (2021). The role of regulatory B cells in kidney diseases. Front Immunol.

[5] Matsushita T (2010). Regulatory B cells (B10 cells) and regulatory T cells have independent roles in controlling experimental autoimmune encephalomyelitis initiation and late-phase immunopathogenesis. J Immunol.

[6] Zouggari Y (2013). B lymphocytes trigger monocyte mobilization and impair heart function after acute myocardial infarction. Nat Med.

[7] Wu L (2019). IL-10-producing B cells are enriched in murine pericardial adipose tissues and ameliorate the outcome of acute myocardial infarction. Proc Natl Acad Sci U S A.

[8] Goodchild TT (2009). Bone marrow-derived B cells preserve ventricular function after acute myocardial infarction. JACC Cardiovasc Interv.

[9] Gu XL (2017). Tim-1^+^ B cells suppress T cell interferon-gamma production and promote Foxp3 expression, but have impaired regulatory function in coronary artery disease. APMIS.

[10] Zhu Z (2018). IL-35 promoted STAT3 phosphorylation and IL-10 production in B cells, but its production was reduced in patients with coronary artery diseases. Hum Immunol.

[11] Douna H (2019). Bidirectional effects of IL-10^+^ regulatory B cells in Ldlr^-/-^ mice. Atherosclerosis.

[12] Tedder TF (2015). B10 cells: a functionally defined regulatory B cell subset. J Immunol.

[13] Kaku H (2014). A novel mechanism of B cell-mediated immune suppression through CD73 expression and adenosine production. J Immunol.

[14] Wasik M (2018). Regulatory B cell phenotype and mechanism of action: the impact of stimulating conditions. Microbiol Immunol.

[15] Ding T (2021). Frontiers of autoantibodies in autoimmune disorders: crosstalk between Tfh/Tfr and regulatory B cells. Front Immunol.

[16] Jia D (2019). Interleukin-35 promotes macrophage survival and improves wound healing after myocardial infarction in mice. Circ Res.

[17] Yan X (2013). Temporal dynamics of cardiac immune cell accumulation following acute myocardial infarction. J Mol Cell Cardiol.

[18] Sabroe I (2003). Selective roles for Toll-like receptor (TLR)2 and TLR4 in the regulation of neutrophil activation and life span. J Immunol.

[19] Tian J (2001). Lipopolysaccharide-activated B cells down-regulate Th1 immunity and prevent autoimmune diabetes in nonobese diabetic mice. J Immunol.

[20] Hahne M (1996). Activated B cells express functional Fas ligand. Eur J Immunol.

[21] Tombor LS (2021). Single cell sequencing reveals endothelial plasticity with transient mesenchymal activation after myocardial infarction. Nat Commun.

[22] Coxon A (1996). A novel role for the beta 2 integrin CD11b/CD18 in neutrophil apoptosis: a homeostatic mechanism in inflammation. Immunity.

[23] Weinmann P (2003). A role for apoptosis in the control of neutrophil homeostasis in the circulation: insights from CD18-deficient mice. Blood.

[24] Atif SM (2019). Protective role of B cells in sterile particulate-induced lung injury. JCI Insight.

[25] Rocha-Resende C (2021). B cells modulate the expression of MHC-II on cardiac CCR2^-^ macrophages. J Mol Cell Cardiol.

[26] Adamo L (2020). Myocardial B cells are a subset of circulating lymphocytes with delayed transit through the heart. JCI Insight.

[27] Adamo L (2018). Modulation of subsets of cardiac B lymphocytes improves cardiac function after acute injury. JCI Insight.

[28] Jiao J (2021). Regulatory B cells improve ventricular remodeling after myocardial infarction by modulating monocyte migration. Basic Res Cardiol.

[29] Renner B (2010). B cell subsets contribute to renal injury and renal protection after ischemia/reperfusion. J Immunol.

[30] Horckmans M (2017). Neutrophils orchestrate post-myocardial infarction healing by polarizing macrophages towards a reparative phenotype. Eur Heart J.

[31] Kim JH (2018). Aged polymorphonuclear leukocytes cause fibrotic interstitial lung disease in the absence of regulation by B cells. Nat Immunol.

[32] Bodhankar S (2013). IL-10-producing B-cells limit CNS inflammation and infarct volume in experimental stroke. Metab Brain Dis.

[33] Burne-Taney MJ (2003). B cell deficiency confers protection from renal ischemia reperfusion injury. J Immunol.

[34] Almishri W (2015). Rapid activation and hepatic recruitment of innate-like regulatory B cells after invariant NKT cell stimulation in mice. J Hepatol.

[35] Ray A (2015). IL-10-independent regulatory B-cell subsets and mechanisms of action. Int Immunol.

[36] Catalán D (2021). Immunosuppressive mechanisms of regulatory B cells. Front Immunol.

[37] Ray A, Dittel BN (2017). Mechanisms of regulatory B cell function in autoimmune and inflammatory diseases beyond IL-10. J Clin Med.

[38] Rafei M (2009). A granulocyte-macrophage colony-stimulating factor and interleukin-15 fusokine induces a regulatory B cell population with immune suppressive properties. Nat Med.

[39] Lecoanet-Henchoz S (1995). CD23 regulates monocyte activation through a novel interaction with the adhesion molecules CD11b-CD18 and CD11c-CD18. Immunity.

[40] Walzog B (1997). Beta2 integrins (CD11/CD18) promote apoptosis of human neutrophils. FASEB J.

[41] Wang L (2021). The dynamic profile and potential function of B-cell subsets during pregnancy. Cell Mol Immunol.

[42] Huang FY (2019). The bifunctional SDF-1-AnxA5 fusion protein protects cardiac function after myocardial infarction. J Cell Mol Med.

[43] Pinto AR (2016). Revisiting cardiac cellular composition. Circ Res.

[44] Sun L (2015). Ex vivo and in vitro effect of serum amyloid a in the induction of macrophage M2 markers and efferocytosis of apoptotic neutrophils. J Immunol.

