# Supplementary material for: B cell subsets contribute to myocardial protection by inducing neutrophil apoptosis after ischemia and reperfusion
Source: JCI Insight. 2024 Feb 22;9(4):e167201. doi: 10.1172/jci.insight.167201 (PMC10967377; doi:10.1172/jci.insight.167201)
Supplement: Supplemental data [file jciinsight-9-167201-s036.pdf]

Reagents and antibodies

FITC-labeled anti-mouse CD45 (Catalog #103108), APC-labeled anti-mouse CD19 (Catalog #115512), PE-labeled anti-mouse CD5 (Catalog #100607), Pacific blue-labeled anti-mouse B220(Catalog #103227), PerCP/Cyanine5.5-labeled anti-mouse CD43(Catalog #143219), Brilliant violet 421-labeled anti-mouse IL10 (Catalog #505021), Brilliant Violet 421-labeled Rat IgG2b, κ Isotype Ctrl (Catalog #400639), PE-labeled anti-mouse CD39 (Catalog #143803), PE-labeled anti-mouse CD73 (Catalog #127205), PE-labeled anti-mouse LAP(TGFβ1) (Catalog #141403), PE-labeled anti-mouse CD45.2(Catalog #109807), FITC anti-mouse CD45.1 (Catalog #110705), purified anti-mouse CD16/32 (Catalog #101302), Brilliant violet 785-labeled anti-mouse Ly6G (Catalog #127645), PE-labeled anti-mouse CD11b (Catalog #101207), PerCP/Cyanine5.5-labeled anti-mouse F4/80 (Catalog #123127), APC-labeled anti-mouse Ly6C (Catalog #128015), Brilliant Violet 510-labeled anti-mouse I-A/I-E (Catalog #107635), APC-labeled anti-mouse CD206 (MMR) (Catalog #141707), Brilliant violet 421-labeled anti-mouse TNFα (Catalog #506327), Brilliant Violet 421-labeled Rat IgG1, κ Isotype Ctrl (Catalog #400429), PE-labeled anti-mouse CD178 (FasL) (Catalog #106605), APC-labeled anti-mouse CD95 (Fas) (Catalog #152603),Brilliant violet 421-labeled anti-mouse PD-L1 (Catalog #124315), APC-labeled anti-mouse CD279 (PD-1) (Catalog #135209), PE-labeled anti-mouse CD253 (TRAIL) (Catalog #109305),PE-labeled anti-mouse CD262 (TRAIL-R2) (Catalog #119905), Brilliant Violet 421-labeled anti-mouse CD23 (Catalog #101621), purified anti-mouse CD23 (Catalog #101602), Ultra-LEAF™ purified anti-mouse CD20 (Catalog #152116), Ultra-LEAF™ purified Rat IgG2b, κ Isotype Ctrl (Catalog #400672), Ultra-LEAF™ purified anti-mouse/human CD45R/B220 (Catalog #103269), Ultra-LEAF™ purified anti-mouse Ly6G (Catalog #127662), Ultra-LEAF™ Purified Rat IgG2a, κ Isotype Ctrl (Catalog #400566), Ultra-LEAF™ purified anti-mouse CD18 (Catalog #101420), Zombie Yellow™ Fixable Viability Kit (Catalog #423103), Precision count Beads (Catalog #424902), MojoSort™ Mouse Pan B-cell Isolation Kit II (Catalog #480088), MojoSort™ Mouse Neutrophil Isolation Kit (Catalog #480058) and Monensin (Catalog #420701) purchased from Biolegend. InVivoMab anti-mouse CD22 (Catalog #BE0011) was purchased from Bio X Cell. DAPI, Percoll, lipopolysaccharide (LPS), collagenase I, collagenase XI, hyaluronidase, and DNase I were purchased from Sigma-Aldrich. Collagenase II was from Worthington. Cell culture medium

and supplements, fetal bovine serum, and phosphate buffer solution (PBS) were from Gibco. Foxp3/Transcription Factor Fixation/Permeabilization kit was from eBioscience. Other reagents are specified below in specific assays.

Generation of bone marrow-chimeric mice

Chimeric mice were generated as previously published (1). In brief, C57BL/6JGpt-$Ptprc^{em1Cin(p.K302E)}$(B6-CD45.1) mice underwent γ-irradiation at a dose of 1150 cGy using a cesium source. Twenty-four hours later, recipients were infused with 1 x 10^7 CD45.2-background donor bone marrow cells. T cells within the donor bone marrow were selectively depleted using the MojoSort™ Mouse CD90.2 Selection Kit (Catalog #480101). For the generation of mice with exclusive IL-10 deficiency in B cells, μMT mice were reconstituted with a bone marrow mixture comprising 80% from μMT and 20% from $IL10^{-/-}$ mice. Control mice received a blend of 80% from μMT and 20% from wild-type mice, while an additional control group received 100% bone marrow from μMT mice. Chimeras were allowed a 2-month period for complete reconstitution of their peripheral lymphoid system before undergoing myocardial I/R. Flow cytometry was employed to confirm chimeric status by detecting the percentage of $CD45.1^+$ and $CD45.2^+$ cells in the peripheral blood.

Spleen B-cell isolation

After euthanizing mice, the spleens were homogenized in a Petri dish to obtain a single-cell suspension. Cell suspensions were filtered through a 70μm cell strainer. Filtered single-cell suspensions were washed twice using Mojosort™ Buffer (Catalog #480017). The cells were adjusted to 10^7/ml in Mojosort™ Buffer. B-cells were isolated using a MojoSort™ PanB-cell Isolation Kit II as described by the manufacturer. The effectiveness of B-cell purification was determined by fluorescence-activated cell sorting (FACS) analysis using APC-labelled anti-mouse CD19. After purification, B-cell purity was over 95%.

Adoptive transfer of B-cells

The adoptive transfer of B cells followed previously protocols (2). Splenic B cells were isolated from a B6/JGpt-$Il10^{em1Cd4885}$/Gpt mouse using the MojoSortTM PanB-cell Isolation Kit

II according to the manufacturer's instructions. Subsequently, $1x10^7$ splenic B cells (in 300µl medium) from *Il10*$^{-/-}$ mice were intravenously injected into μMT mice, both before and 2 days after myocardial I/R. The reconstituted status was confirmed by flow cytometry, assessing the percentage of B220+CD19+ B cells in both the spleen and heart.

In vivo blockade of FCER2A

Mice were intraperitoneally injected with purified anti-mouse CD23 (150µg per mouse) immediately after I/R. the vehicle group received corresponding isotype i.p. injection.

Isolation of bone marrow neutrophils and macrophages

Bone marrow cells were flushed out from both the femur and the tibia. Cell suspensions were filtered through a 70µm cell strainer and washed with PBS. For neutrophil isolation, the filtered cells were adjusted to 10^7/ml in Mojosort™ Buffer. Neutrophils were isolated using a MojoSort™ Mouse Neutrophil Isolation Kit as described by the manufacturer. Freshly isolated neutrophils were cultured in X-VIVO20™ serum-free medium (Lonza).

For macrophage isolation, the filtered bone marrow cells were collected by centrifugation at 400 × g for 5 minutes and the red blood cells were eliminated by Red Blood Lysis Buffer as per the manufacturer's recommendation. Cell pellets were washed twice with DMEM. Bone marrow cells were cultured in DMEM supplemented with 10% FBS, 1% L-glutamine, 55 µM 2-mercaptoethanol, and 20 ng/ml macrophage colony-stimulating factor (# 51112-MNAH, SinoBiological) for 7 days to induce macrophage differentiation.

Primary neonatal murine cardiomyocyte preparation and culture

Primary neonatal murine cardiomyocyte culture was prepared as previously described with minor modifications.(3) Briefly, 1-3 days old neonatal mice were sacrificed and the beating hearts were immediately extracted, washed with PBS containing 20nM 2,3-butanedinone monoxime (BDM), and minced into small pieces. The tissue block was incubated with gentle agitation at 4℃ in 10ml isolation buffer (0.25% trypsin solution with EDTA and 20mM BDM). Following overnight incubation, the digestion buffer (1.5mg/ml collagenase II and 20mM BDM in L15 culture medium) replaced the supernatant. Cardiac

tissue fragments continued to be incubated at 37℃ with gentle agitation at 20rpm for 20-30min. Tissue fragments were gently triturated using a pre-wetted 10ml cell-culture pipette about 10-20 times. Let larger tissue fragments sediment and transfers supernatant containing suspended cells into a fresh conical tube through cell-strainer. The undigested tissue fragments were re-suspended in 5ml digestion buffer and incubated for an additional 5-10 minutes at 37℃ with gentle agitation. Then, cell suspensions were collected and filtered through a 70μ cell strainer. Filtered single-cell suspensions were centrifuged at 200g for 5 minutes. After removing the supernatant, the cell pellet was re-suspended in 10ml DMEM complete growth medium. Cells were plated into a cell culture dish and incubated. After 1hour, non-adherent cardiomyocytes were transferred into collagen-coated 12-well cell culture dishes with a density of approximately 1.5×10^5 cells per cm^2. The medium was replaced every 2-3 days with fresh medium. The primary cultures were maintained till the cultures attained ~80%.

Hypoxia and reoxygenation model

Neonatal murine cardiomyocytes were subjected to hypoxia/reoxygenation as described previously (4). The cell medium was replaced with glucose- and serum-free DMEM(Gibco). The primary cardiomyocytes were cultured in an oxygen-deficient environment (1%O2, 5%CO2, and 94%N2) for 6 hours at 37℃. Then, the cell medium was replaced with DMEM complete medium. The cells continued to be cultured at 95% air, and 5% CO2 with or without B-cell (1×10^6/well) coculture for another 16 hours. Cell apoptosis was characterized by FITC Annexin V Apoptosis Detection Kit (#556547, BD Pharmingen) following the manufacturer's protocols.

Echocardiography

Echocardiography analysis was performed 14 days after myocardial ischemia and reperfusion. After mice were anesthetized by inhalation of 2% isoflurane, transthoracic echocardiography was performed using a Vevo3100 ultrasound machine (VisualSonics, Fujifilm). Measurements were performed at the midpapillary level from well-aligned M-mode images from the parasternal short axis view.

Histology/scoring

Hearts were isolated, and retrogradely perfused with a cardioplegic solution to induce diastolic arrest. Then, specimens were fixed in 4% paraformaldehyde for 48 hours and embedded in paraffin, and 4μm-thick sections were cut and mounted on the positively charged glass slides. The slices were stained with hematoxylin and eosin (Servicebio) according to the standard protocols and observed by light microscopy for changes in histology. A scoring system was used to evaluate the histological myocardial damage based on the modified scoring system described previously(5). Briefly, myocardial damage including interstitial edema, myofibre degeneration, and subendocardial hemorrhage was graded as to their severity (0 = no damage, 1 = mild, 2 = moderate, and 3 = marked) and distribution (0 = no damage, 1 = focal damage, 2 = multifocal damage, 3 = diffuse damage). A mean score for each variable was determined for each heart, and a group mean score was calculated.

Apoptosis in the adult heart was assayed with the DeadEnd Fluorometric TUNEL system (Promega) according to the manufacturer's protocol. Five randomly selected fields of view were observed and TUNEL-positive cells of each section were counted under an inverted fluorescence microscope (Leica).

For Masson's trichrome staining, slices were dyed with Masson Trichrome (Solarbio) according to manufacturer' protocol. The infarct size was measured at the level of the mid-papillary heart muscles. The images were analyzed using Adobe Photoshop software 2021. Values are represented as the proportion of collagen normalized to the left ventricular area.

For immunofluorescence of heart slice, primary antibodies used were anti-mouse Ly6G rabbit polyclonal antibody (Servicebio, 1:200) and anti-mouse B220 monoclonal rat antibody (1:50). Secondary antibodies used were Alexa Fluor® 488-conjugated goat anti-rabbit antibody and Cy3-conjugated AffiniPure goat anti-rat antibody (1:1000, Servicebio). DAPI (1:5,000) was used to counter-stain the nuclei. Representative images of each section were captured with an inverted fluorescence microscope (Leica).

Total RNA extraction and qPCR

Total RNA was isolated by using Quick-RNA Kits according to the manufacturer's recommendation (ZYMO Research) and converted into cDNA using the ReverTra Ace™ qPCR

RT Kit (TOYOBO life science). The mRNA levels of *Il6*, *Il1b*, *Nlrp3*, *Aim2*, *Il10*, *Tgfb1*, *Ccl2*, *Cxcl1*, *Ccl7*, *Cxcl10*, *Ccl12*, and *Atcb* were determined with QuantiFast SYBR® Green PCR Kit (Qiagen). The quantification of the target mRNA expression was done with ΔΔ method using *Atcb* mRNA as reference. The primers for qPCR were listed in the following Table.

Table. Primers used in this study.

Target gene	Forward primer	Reverse primer
Il1b	GCAACTGTTCCTGAACTCAACT	ATCTTTTGGGGTCCGTCAACT
Il6	TAGTCCTTCCTACCCCAATTTCC	TTGGTCCTTAGCCACTCCTTC
Nlrp3	ATTACCCGCCCGAGAAAGG	TCGCAGCAAAGATCCACACAG
Aim2	GTCACCAGTTCCTCAGTTGTG	CACCTCCATTGTCCCTGTTTTAT
Il10	GCTCTTACTGACTGGCATGAG	CGCAGCTCTAGGAGCATGTG
Tgfb1	CTCCCGTGGCTTCTAGTGC	GCCTTAGTTTGGACAGGATCTG
Ccl2	TAAAAACCTGGATCGGAACCAAA	GCATTAGCTTCAGATTTACGGGT
Ccl12	ATTTCCACACTTCTATGCCTCCT	ATCCAGTATGGTCCTGAAGATCA
Ccl7	CCACATGCTGCTATGTCAAGA	ACACCGACTACTGGTGATCCT
Cxcl1	CTGGGATTCACCTCAAGAACATC	CAGGGTCAAGGCAAGCCTC
Cxcl10	CCAAGTGCTGCCGTCATTTTC	GGCTCGCAGGGATGATTTCAA
Actb	GTGACGTTGACATCCGTAAAGA	GCCGGACTCATCGTACTCC

Bulk RNA sequencing

Each group of mice were subject to myocardial I/R. After 24 hours, hearts were collected for RNA extraction. Total RNA was isolated and purified as described above. The RNA amount and purity of each sample were quantified using NanoDrop ND-1000 (NanoDrop, Wilmington, DE, USA). The RNA integrity was assessed by Bioanalyzer 2100 (Agilent, CA, USA) with RIN number >7.0, and confirmed by electrophoresis with denaturing agarose gel. Poly (A) RNA is purified from 1μg total RNA using Dynabeads Oligo (dT)25-61005 (Thermo Fisher, CA, USA) using two rounds of purification. Then the poly(A) RNA was fragmented into small pieces using Magnesium RNA Fragmentation Module (NEB, USA) for 94℃ 5-7min. Then the cleaved RNA fragments were reverse-transcribed to create the cDNA by SuperScript™ II Reverse Transcriptase (Invitrogen, USA), which were next used to synthesize U-labeled second-stranded DNAs with E. coli DNA polymerase I (NEB, USA), RNase H (NEB, USA) and dUTP Solution (Thermo Fisher, USA）. An A-base is then added to the blunt ends of each strand, preparing them for ligation to the indexed adapters. Each adapter contains a T-base

overhang for ligating the adapter to the A-tailed fragmented DNA. Single- or dual-index adapters are ligated to the fragments, and size selection was performed with AMPureXP beads. After the heat-labile UDG enzyme (NEB, USA) treatment of the U-labeled second-stranded DNAs, the ligated products are amplified with PCR by the following conditions: initial denaturation at 95℃ for 3 min; 8 cycles of denaturation at 98℃ for 15 sec, annealing at 60℃ for 15 sec, and extension at 72℃ for 30 sec; and then final extension at 72℃ for 5 min. The average insert size for the final cDNA library was 300±50 bp. At last, we performed the 2×150bp paired-end sequencing (PE150) on an Illumina Novaseq™ 6000 (LC-Bio Technology CO., Ltd., Hangzhou, China) following the vendor's recommended protocol.

The fastp software was used to remove the reads that contained adaptor contamination, low-quality bases, and undetermined bases with default parameters. The sequence quality was also verified using fastp. We used HISAT2 to map reads to the reference genome of Mus musculus GRCm38. The mapped reads of each sample were assembled using StringTie with default parameters. Then, all transcriptomes from all samples were merged to reconstruct a comprehensive transcriptome using gffcompare. After the final transcriptome was generated, StringTie was used to perform expression level for mRNAs by calculating FPKM (FPKM = [total_exon_fragments/mapped_reads(millions)×exon_length(kB)]). The differentially expressed genes (DEGs) were selected with fold change > 2 or fold change < 0.5 and with a parametric F-test comparing nested linear models (p-value < 0.05) by R package edgeR. Functional enrichment analysis, predominantly based on the Gene Ontology (GO) and Kyoto Encyclopedia of Genes and Genomes (KEGG) database, was used to determine the significant function and pathway of DEGs. Gene Set Enrichment Analysis (GSEA) was performed via R package ClusterProfiler with 50 hallmark gene sets that were downloaded from the R msigdbr package. R package GseaVis was used to visualize GSEA enrichment results.

Single-cell RNAseq

The single-cell RNA sequencing (scRNA-seq) data in this study were downloaded from the Array Express repository (EMTAB-9816). Raw Illumina data were processed using CellRange 6.0.1; data quality control, normalization, clustering, and marker analysis were performed using Seurat software package version 4.0.6 in R version 4.1.0. Data from 2 samples

(sham and day 3 post-MI) were merged. The following criteria were applied to each sample cell: feature gene number between 200 and 5000, UMI counts >1000, mitochondrial gene percentage<10%. 3671 cells were left for further analysis. Read counts were normalized using the 'LogNormalize' method. The percentage of mitochondrial reads was also regressed out. Data were scaled using "ScaleData", and variable genes were detected using the "FindVariableFeatures" function with default parameters (selection method 'vst', nfeatures 2000). Dimensionality reduction was used to explore transcriptional heterogeneity and for clustering. Specifically, PCA and UMAP were used for dimensionality reduction. The top 20 principal components (PCs) were used as input for the "RunUMAP" function, 20 PCs for "FindNeighbours" and resolution 0.5 to "FindClusters". "FindAllMarkers" were employed to identify cluster-conserved (marker) genes. "DotPlot" and "VlnPlot" functions were used for plotting gene expression data.

Ligand–receptor prediction analysis

We used the CellChat R package to infer the cell-cell interaction network based on the "Cell-Cell Contact" sub-library of the Cellchat database as previously described(6). Briefly, CellChat input file was exact from Seurat object and preprocessed using "identifyOverExpressedGenes", "identifyOverExpresssedInteractions", and "projectData" with default parameters. The "computeCommunProb", "computeCommunProbPathway", and "aggregateNet" were applied to infer the cellular communication networks. Graphs were generated using the "netVisual_chord_gene" function.

Reference

1. Fillatreau S, Sweenie CH, McGeachy MJ, Gray D, Anderton SM. B cells regulate autoimmunity by provision of IL-10. Nature immunology. 2002;3(10):944-50.
2. Nishida A, Lau CW, Mizoguchi E, Mizoguchi A. Regulatory B cells in mouse models of intestinal inflammation. Methods in molecular biology. 2014;1190:227-41.
3. Ehler E, Moore-Morris T, Lange S. Isolation and culture of neonatal mouse cardiomyocytes. J Vis Exp. 2013(79).
4. Huang FY, Xia TL, Li JL, Li CM, Zhao ZG, Lei WH, et al. The bifunctional SDF-1-AnxA5 fusion protein protects cardiac function after myocardial infarction. J Cell Mol Med. 2019;23(11):7673-84.
5. Chen E, Chen C, Niu Z, Gan L, Wang Q, Li M, et al. Poly(I:C) preconditioning protects the heart

against myocardial ischemia/reperfusion injury through TLR3/PI3K/Akt-dependent pathway. Signal Transduct Target Ther. 2020;5(1):216.
6. Jin S, Guerrero-Juarez CF, Zhang L, Chang I, Ramos R, Kuan CH, et al. Inference and analysis of cell-cell communication using CellChat. Nat Commun. 2021;12(1):1088.

A

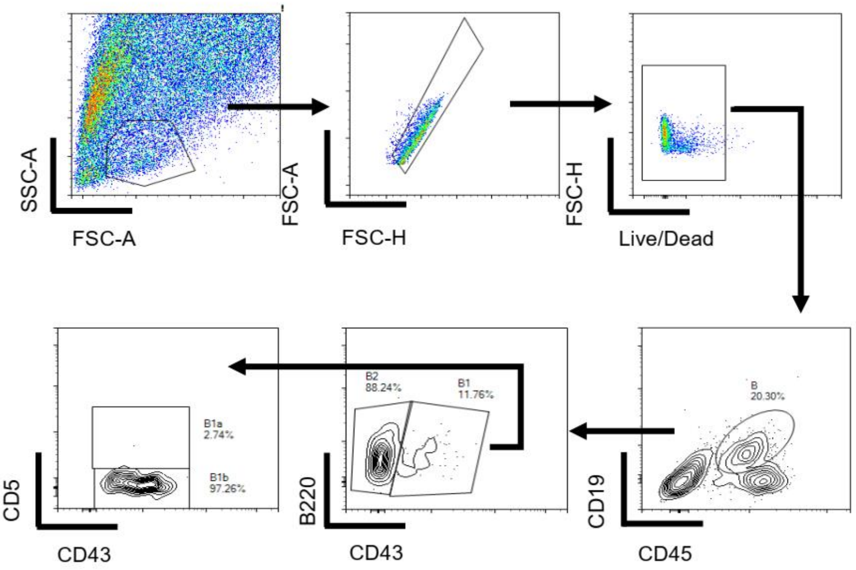

B

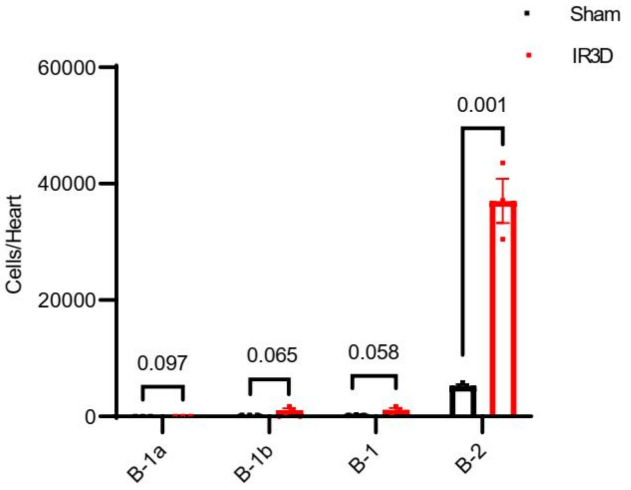

Supplemental Figure 1. The infiltration of B-cell subsets into the heart after myocardial I/R. **(A)** Gate strategies using CD43 distinguishing B1 and B2 and **(B)** quantification of the number of B-cell subsets in the heart. The experiments were independently replicated twice. I/R, ischemia and reperfusion. Differences between the two groups were compared by unpaired t-test.

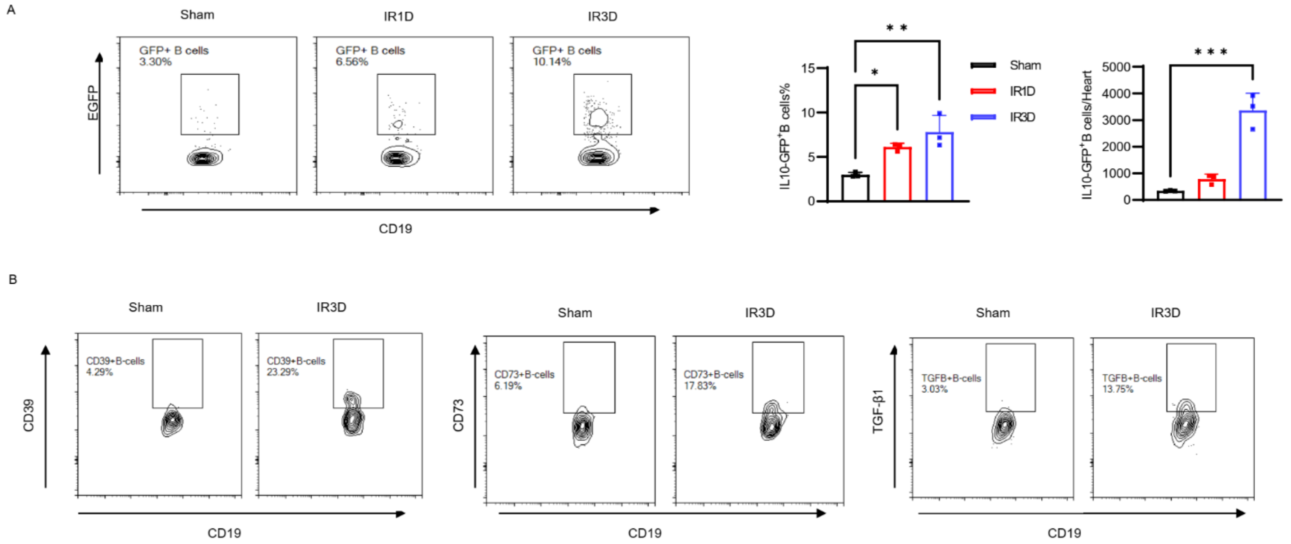

Supplemental Figure 2. The infiltration of regulatory B-cells heart after I/R. **(A)** Representative images for flow cytometric analysis and quantification of percentage and the number of myocardial EGFP^{+} B-cells 1 and 3 days after myocardial I/R in Vert-X mice. **(B)** Representative expression of CD39， CD73 and TGF-β1 by myocardial B-cells 3 days after I/R. *p<0.05, **<0.01, ***<0.001. Statistical tests were ANOVA test followed by Tukey post hoc tests.

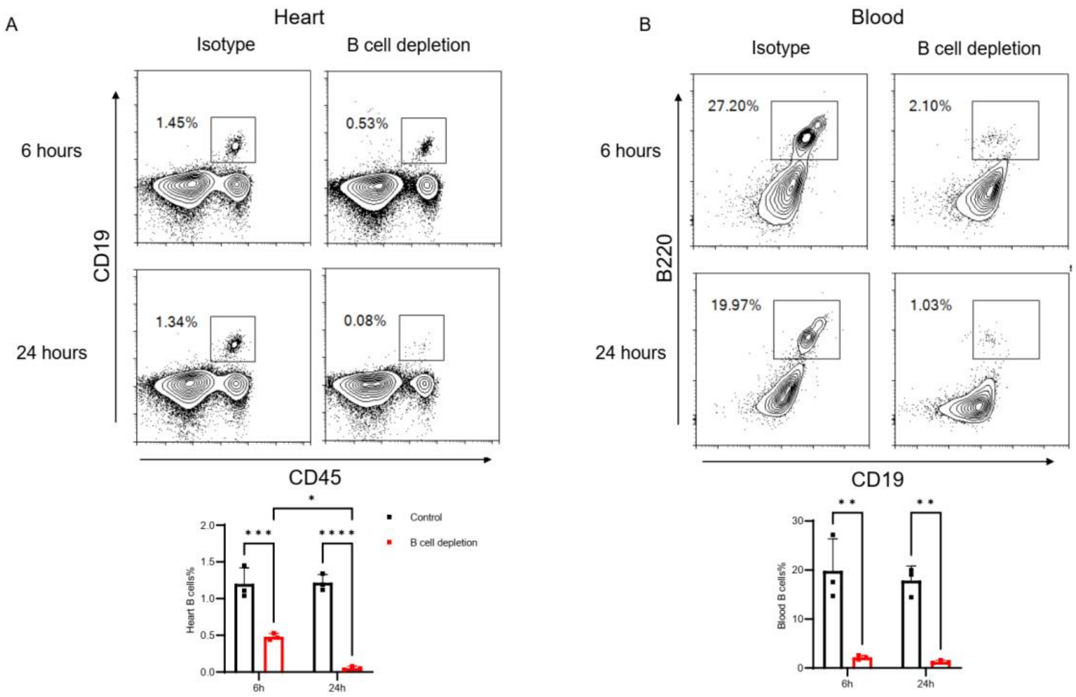

Supplemental Figure 3. Anti-CD20-mediated depletion of B cells in the murine **(A)** heart and **(B)** blood. C57BL6J mice were intravanousely injected with isotype control or anti-CD20 antibody (200ug per mouse), and blood and heart were analyzed via flow cytometry 6 and 24 hours later to confirm B cell depletion. *P<0.05, ***P<0.001. The experiments were independently replicated twice. Statistical tests were ANOVA test followed by Tukey post hoc tests.

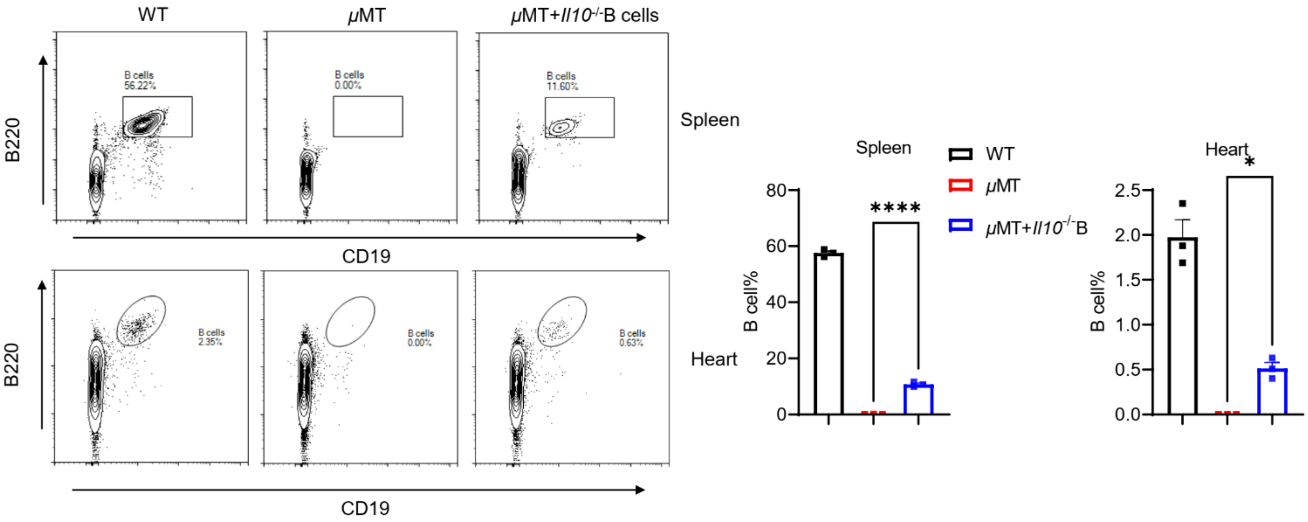

Supplemental Figure 4. Adoptive transfer of *Il10*$^{-/-}$ B cells to μMT mice. A total of 1x10^7 spleen B-cells from IL10$^{-/-}$ mice were transferred into μMT mice before and 2 days after myocardial I/R. Wild-type, μMT and, μMT mice transferred with *Il10*$^{-/-}$B-cells were subjected to myocardial I/R. ****$p<0.0001$, *$p<0.05$. WT, wild-type.Statistical tests were ANOVA test followed by Tukey post hoc tests.

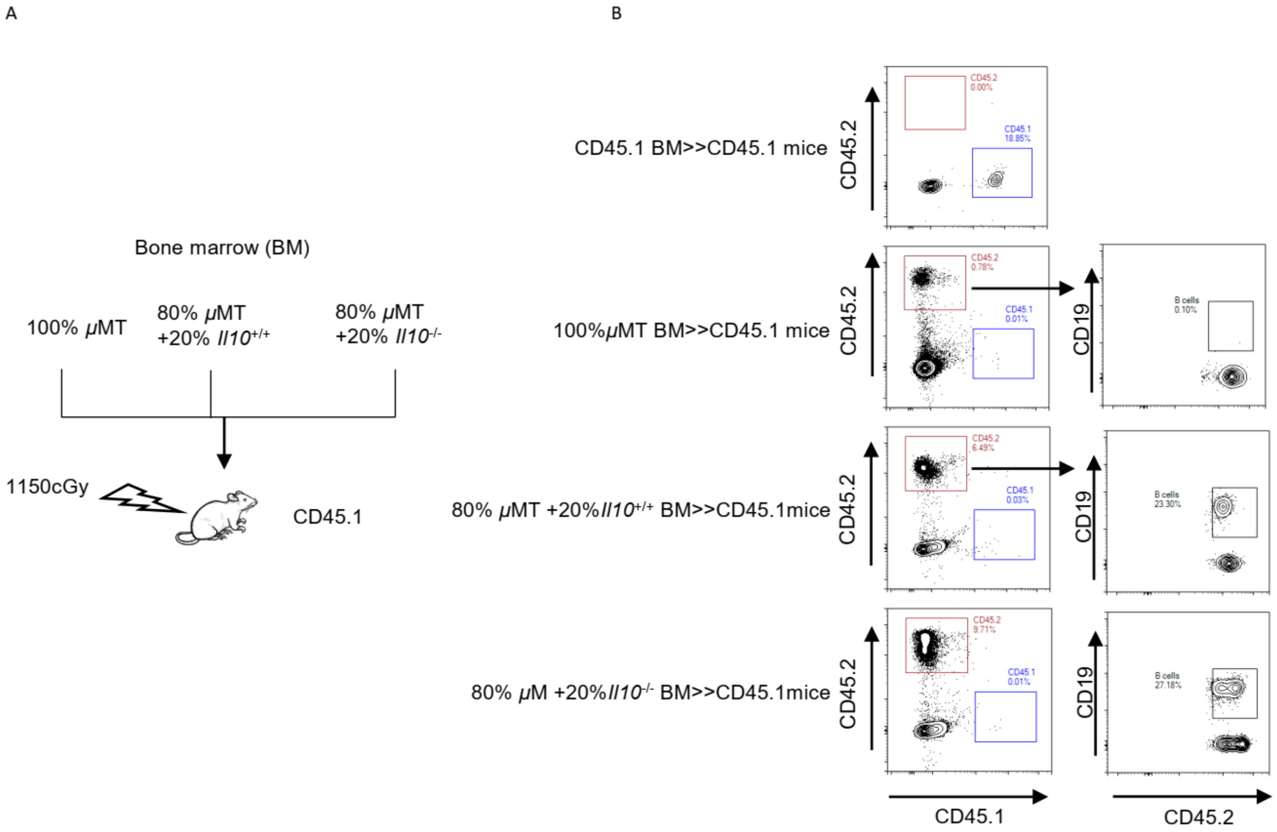

Supplemental Figure 5. Generation of chimeric mice. **(A)** Bone marrow-chimeric mice with gene deficiency restricted to B cells were generated by reconstituting CD45.1^{+} B6 mice with μMT bone marrow with or without bone marrow from *IL10*$^{+/+}$ or *IL10*$^{-/-}$ mice, and chimeras were left to fully reconstitute their peripheral lymphoid systems for 2months, and **(B)** blood were analyzed via flow cytometry 2 months.

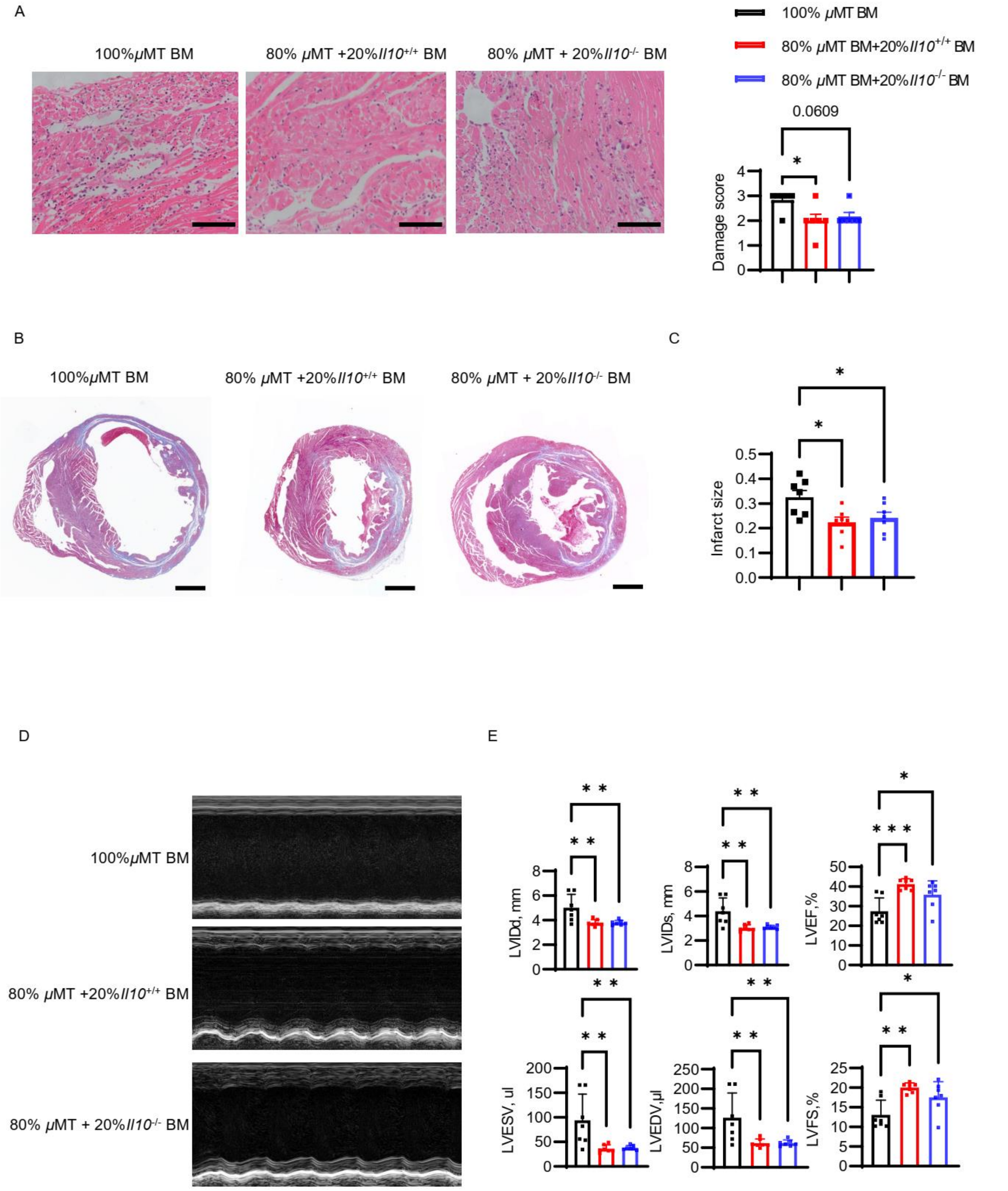

Supplemental Figure 6. Evaluation of myocardial ischemia-reperfusion injury in a bone marrow chimeric system with B cell-specific gene deficiency. Two months post-reconstitution, chimeric mice receiving either completely deficient B cells or 20% B cells with or without *Il10* knockout bone marrow underwent myocardial ischemia-reperfusion (I/R). **(A)** Representative images of HE-stained heart slices in each group 1 days after myocardial I/R (scale bar=100μm) and corresponding damage scores quantifying tissue injury. *P<0.05 (n=6 mice per group). **(B)** Representative Masson trichrome staining of heart slices and **(C)** quantification of infarct size after myocardial I/R in each group. **(D)** Representative M-mode echocardiography

images of each group 14 days after I/R. (E) Measurements of cardiac function and cardiac size in each group 14 days after I/R. *$P<0.05$, **$P<0.01$, and ***$P<0.001$. Statistical tests were ANOVA test followed by Tukey post hoc tests.

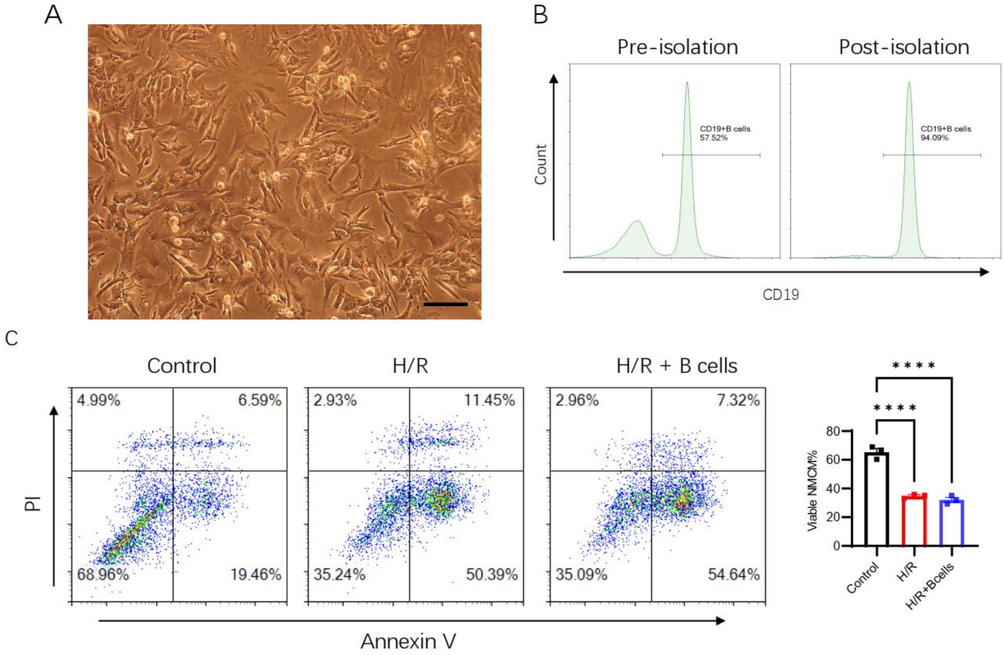

Supplemental Figure 7. B-cells did not influence the viability of neonatal murine cardiomyocytes that were subjected to hypoxia/reoxygenation (H/R) in vitro. **(A)** Representative images of neonatal murine cardiomyocytes 3 days following primary isolation. **(B)** The purity of B-cells was determined by flow cytometry before and after spleen B cells were negatively selected using magnetically activated cell sorting. **(C)** NMCMs that were cocultured with B cell or alone were treated with H/R or normoxia, as described in Materials and Methods. Representative flow cytometry images for the detection and quantification of apoptotic cardiomyocytes. ****$p<0.000$. Statistical tests were ANOVA test followed by Tukey post hoc tests.

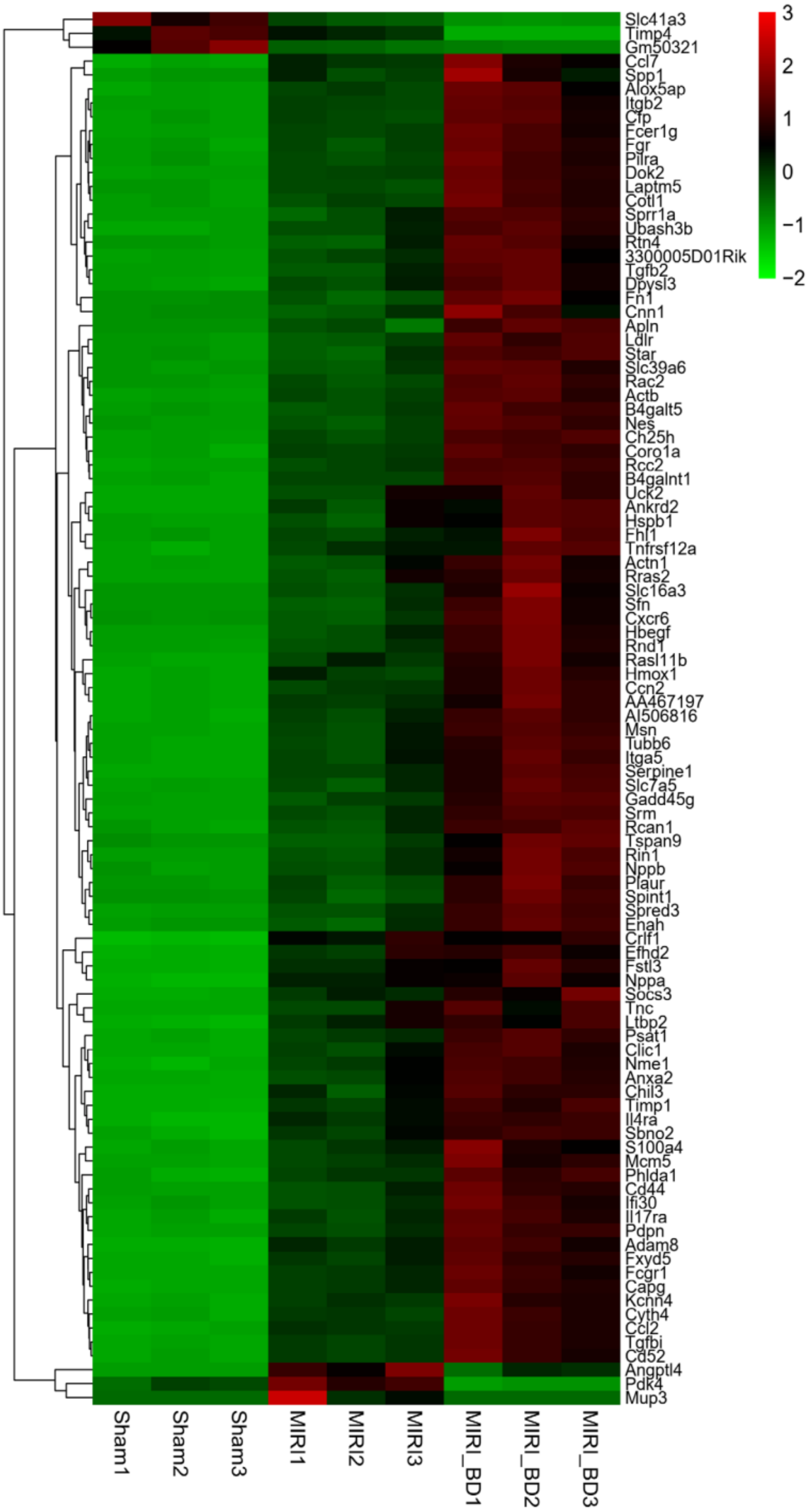

Supplemental Figure 8. the heatmap showing the mRNA expression of top 100 DEGs of hearts among the three groups. DEG, differentially expressed gene; BD, B-cell depletion.

MIRI vs. Sham

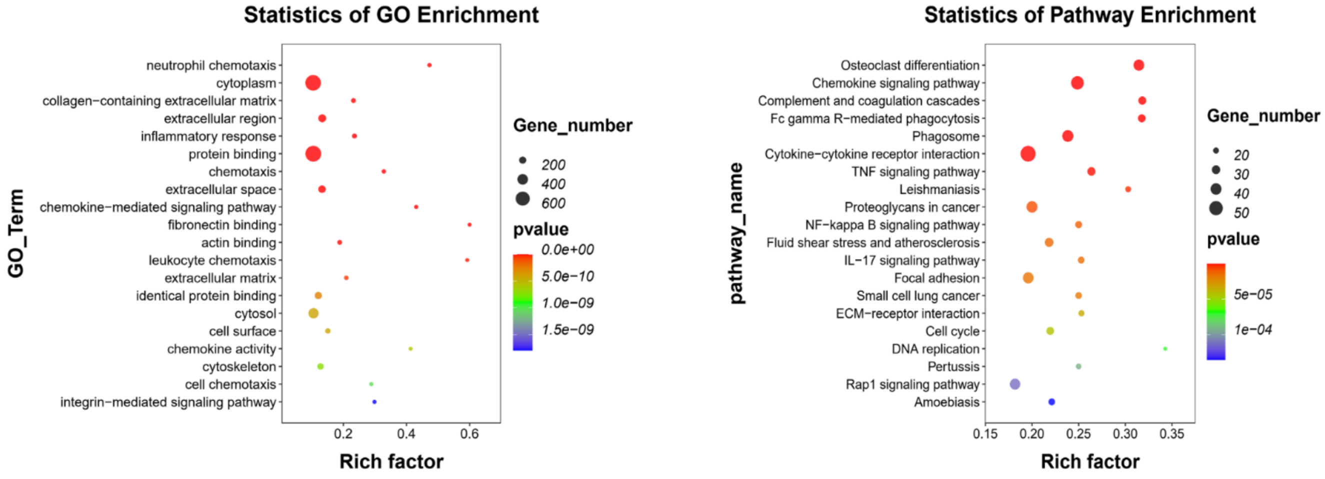

Supplemental Figure 9. Gene enrichment analysis. **(A)**Top 20 gene ontology and **(B)** kyoto encyclopedia of genes and genomes enrichment analyses of heart DEGs between MIRI and Sham group. Y-axis represents different pathways. X-axis represents the percentage that each function group gene accounts for the total genes, respectively. MIRI, myocardial ischemia and reperfusion injury.

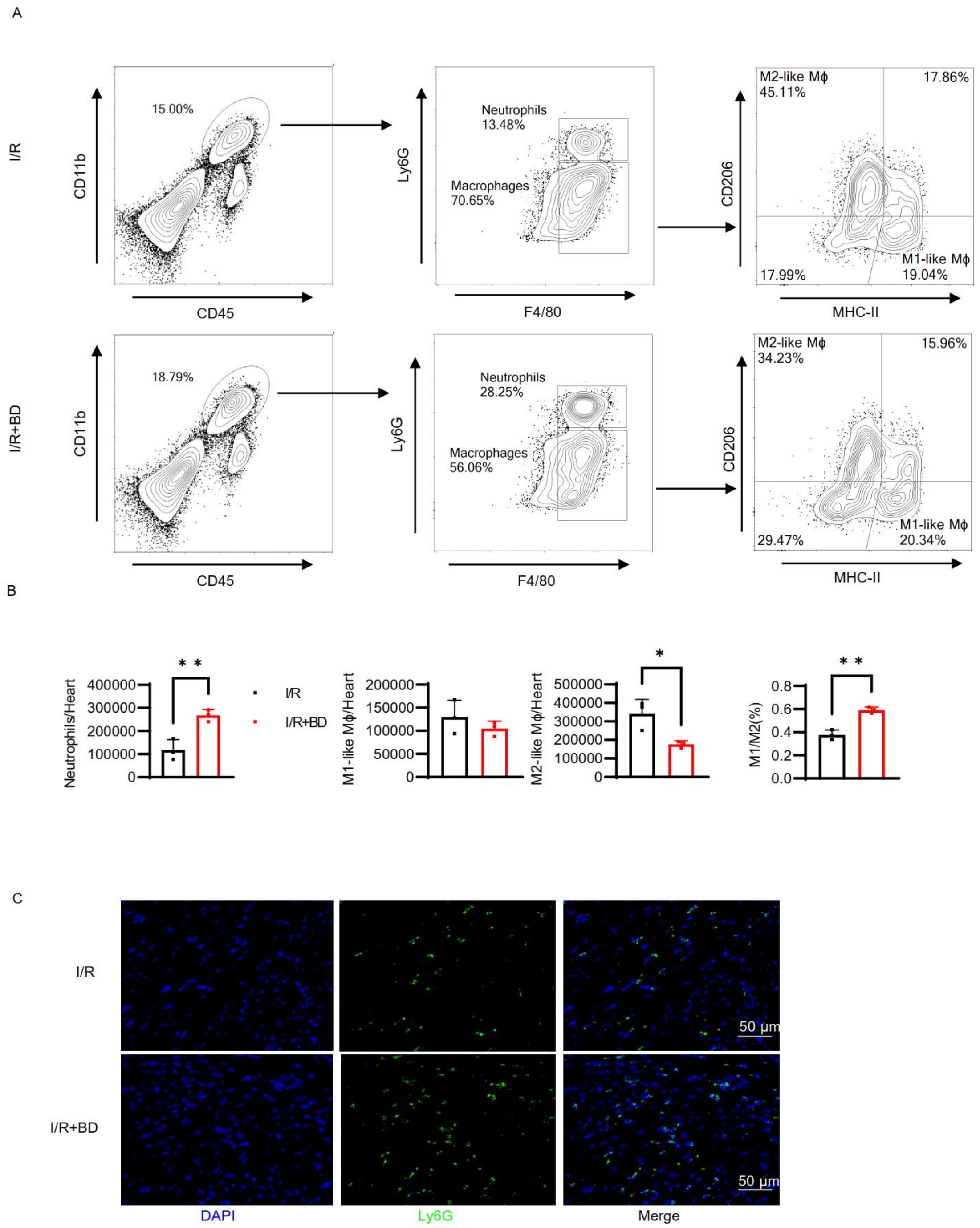

Supplemental Figure 10. Selective B-cell depletion exacerbates the early-phase inflammatory response following myocardial I/R. Mice subjected to myocardial I/R, with or without B-cell depletion (BD), had their hearts harvested and digested 24 hours post-I/R. **(A)** Illustration of gate strategies and **(B)** quantification of neutrophils and M1/M2-like macrophages in the heart. **(C)** Representative immunofluorescence images of Ly6G in heart sections from the two experimental groups (scale bar=50μm). *P<0.05, **P<0.01. The experiments were independently replicated twice. Differences between the two groups were compared by unpaired t-test.

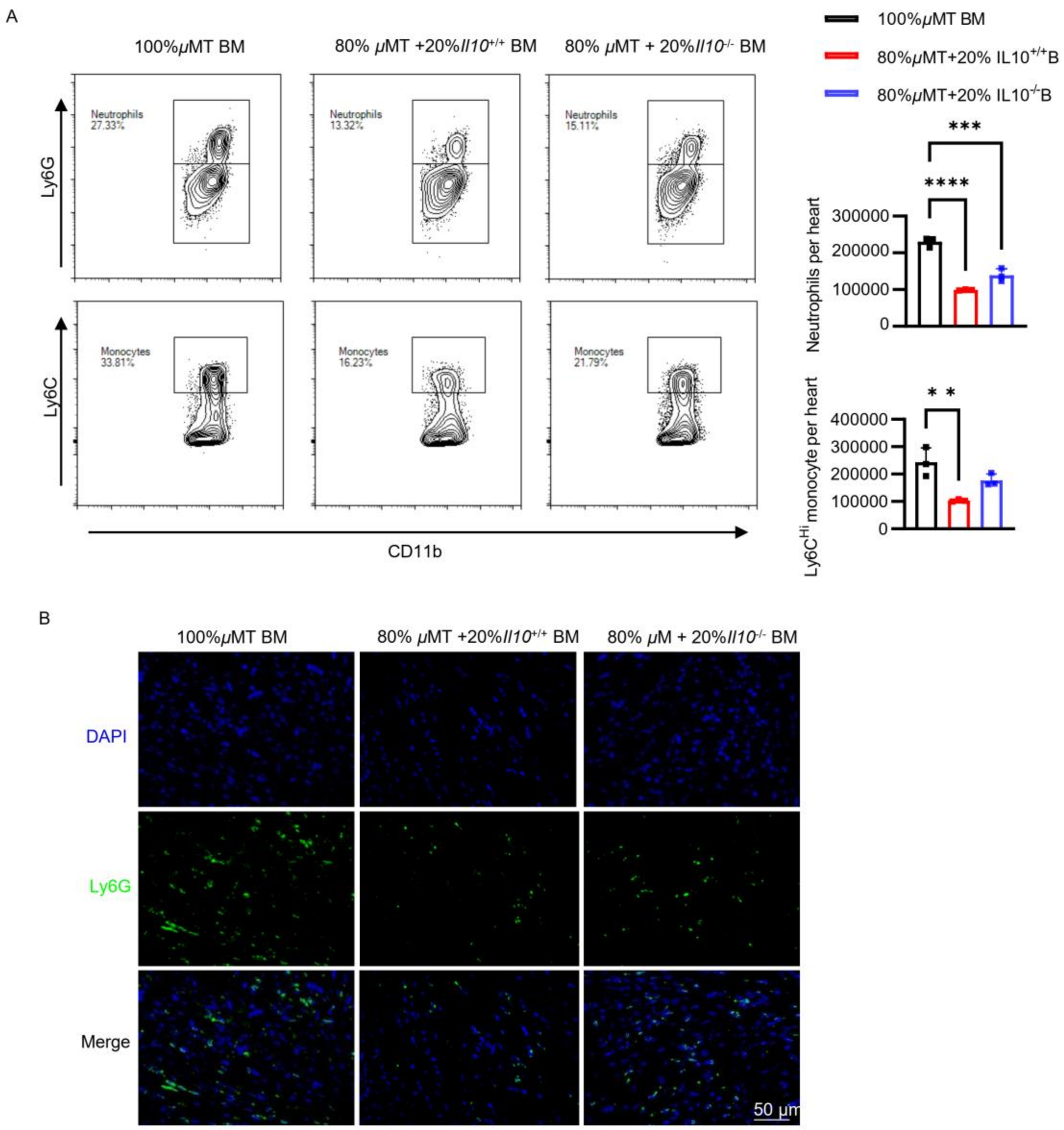

Supplemental Figure 11. Evaluation of inflammatory response following myocardial I/R in CD45.1 mice reconstituted with a hybrid bone marrow composition (80% from *μ*MT mice and 20% from both *Il10*$^{+/+}$ and *Il10*$^{-/-}$ mice). Flow cytometry **(A)** and immunofluorescence **(B)** analyses illustrate the observed reduction in neutrophil presence (scale bar=50μm). **P<0.01, ***P<0.001 The experiments were independently replicated twice. BM, bone marrow. Statistical tests were ANOVA test followed by Tukey post hoc tests.

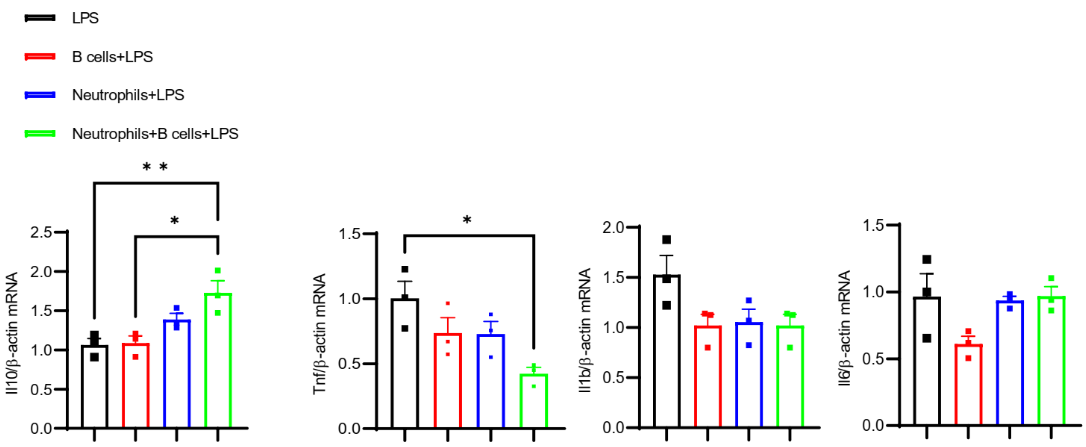

Supplemental Figure 12. Cytokine mRNA levels of BMDM. BMDM was treated with LPS (100ng/ml) for 12 hours in presence of B-cells or/and neutrophils. After washing out the B-cells and neutrophils in suspension, the adherent BMDM was detected for cytokine mRNA expression by qPCR. *$p<0.05$, **$p<0.01$. Statistical tests were ANOVA test followed by Tukey post hoc tests.

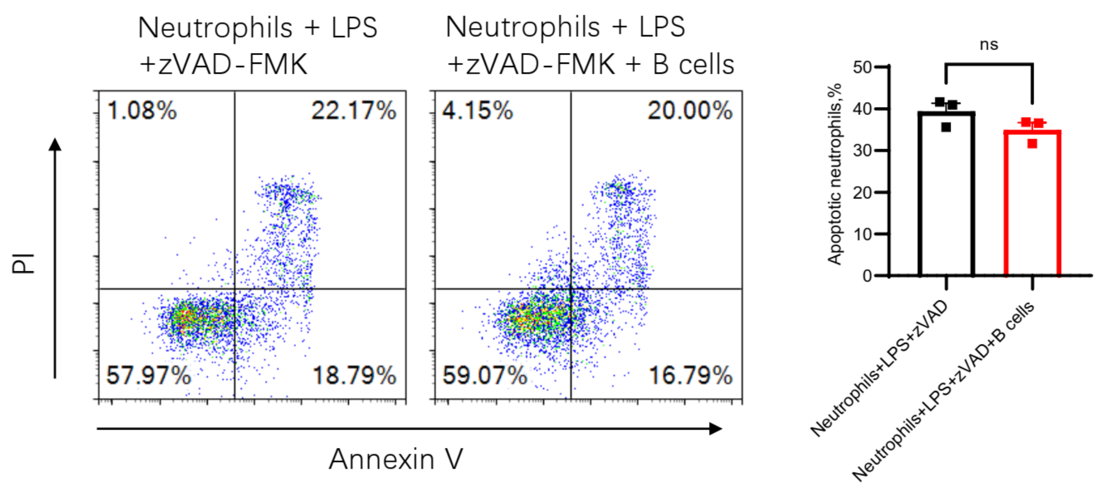

Supplemental Figure 13. B-cell-induced neutrophil apoptosis was blocked by pan-caspase inhibitor, zVAD-FMK. Neutrophils pretreated with zVAD-FMK overnight were cocultured with or without B cells upon LPS (100ng/ml) stimulation overnight. The percentage of apoptotic neutrophils was detected by flow cytometry. The experiments were independently replicated twice. Differences between the two groups were compared by unpaired t-test.

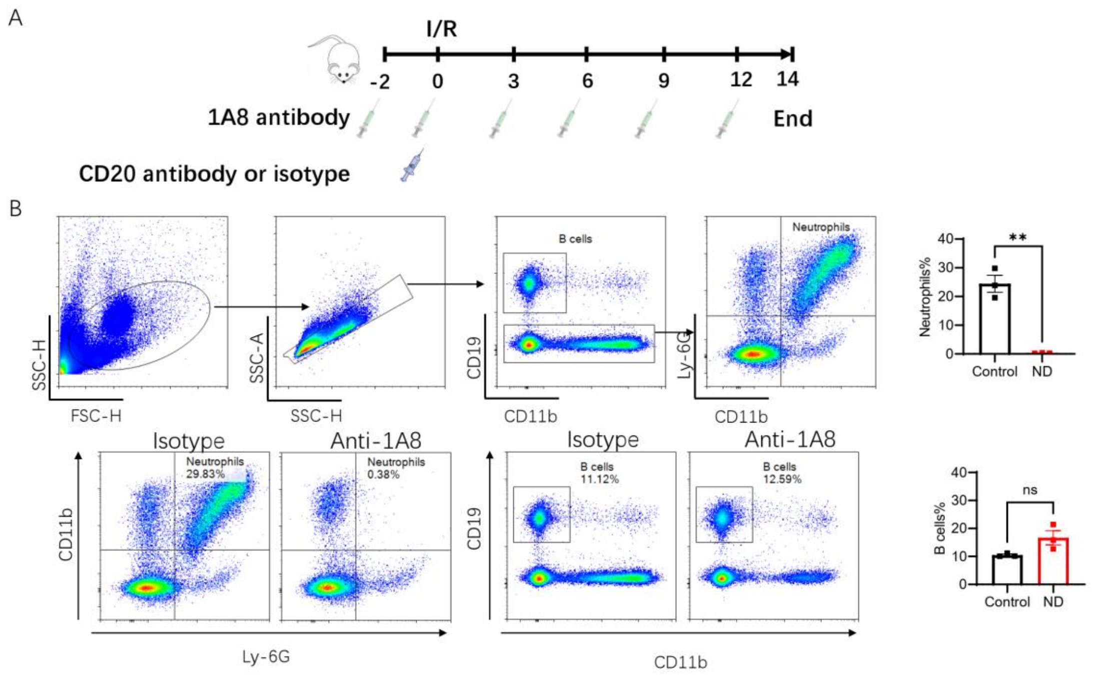

Supplemental Figure 14. circulating neutrophils and B cells in neutrophil-depleted mice. **(A)** Experimental protocol for treatment with neutrophil-depleting antibody (1A8) or isotypewith or without B-cell depletion using CD20 antibody. **(B)** Gating strategy used for the quantification of neutrophils% and B-cells% in blood 1 days after myocardial ischemia and reperfusion. **P<0.01. The experiments were independently replicated twice. Differences between the two groups were compared by unpaired t-test.

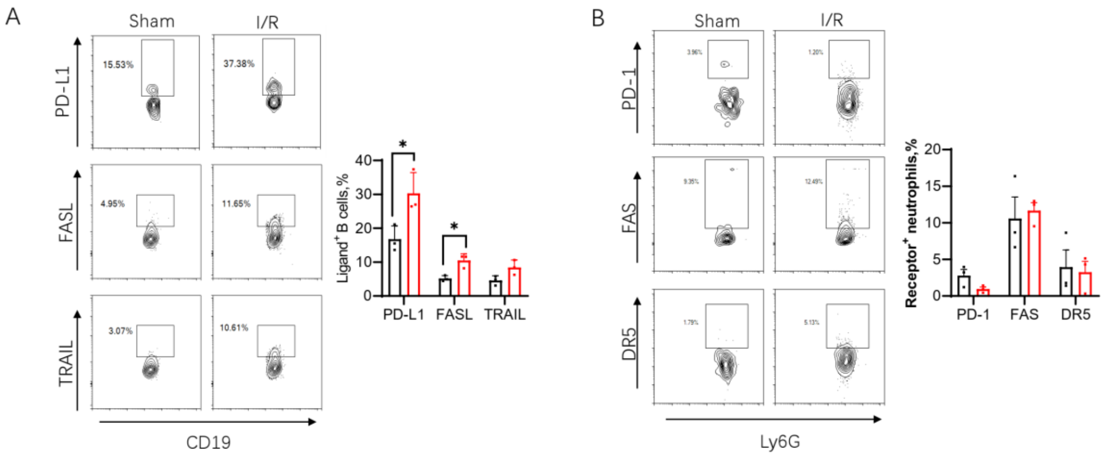

Supplemental Figure 15. Heart B-cells and neutrophils were screened for expression of traditional death receptors and receptor-ligands 3 days after I/R, respectively. **(A)** the expression of PD-L1, FASL and TRAIL by heart B-cells. **(B)** the expression of PD-1, FAS and DR5 by heart neutrophils. *P<0.05. The experiments were independently replicated twice. Differences between the two groups were compared by unpaired t-test.
